# LYM2 mediates chitin-induced plasmodesmal flux reduction in *Populus* x *canescens*

**DOI:** 10.3389/fpls.2026.1879338

**Published:** 2026-07-13

**Authors:** Mo Awwanah, Merle Reinhold, Greta Niemann, Mascha Muhr, Kerstin Schmitt, Oliver Valerius, Andrzej Majcherczyk, Dennis Janz, Elena Petutschnig, Volker Lipka, Thomas Teichmann

**Affiliations:** 1 Department of Plant Cell Biology, Albrecht-von-Haller-Institute of Plant Sciences, University of Göttingen, Göttingen, Germany; 2 Research Center for Genetic Engineering, National Research and Innovation Agency (BRIN), Bogor, Indonesia; 3 Department of Molecular Microbiology and Genetics, Institute for Microbiology and Genetics, University of Göttingen, Göttingen, Germany; 4 Service Unit LCMS Protein Analytics, Göttingen Center for Molecular Biosciences (GZMB), University of Göttingen, Göttingen, Germany; 5 Department of Molecular Wood Biotechnology and Technical Mycology, University of Göttingen, Göttingen, Germany; 6 Department of Forest Botany and Tree Physiology, Büsgen-Institute, University of Göttingen, Göttingen, Germany; 7 Central Microscopy Facility of the Faculty of Biology & Psychology, University of Göttingen, Göttingen, Germany; 8 Department of Biology, University of Graz, Graz, Austria

**Keywords:** alternative splicing, chitin perception, LYM2, LysM-RLP, plasmodesmal flux, poplar

## Abstract

**Introduction:**

Lysin motif domain-containing glycosylphosphatidyl inositol-anchored protein 2 (LYM2) is a GPI-anchored LysM receptor-like protein (LysM-RLP) that harbors three extracellular LysM-domains and a GPI moiety at the C- terminus. In rice, the LYM2 homolog CEBiP is part of the canonical chitin receptor complex mediating chitin triggered MAPK activation and ROS burst. In the annual plant *Arabidopsis thaliana*, AtLYM2 is specifically involved in chitin-mediated plasmodesmal flux regulation. The aim of this study is to analyse components of chitin perception in the perennial plant poplar and to explore if LYM2 proteins in dicots are generally involved in chitin mediated PD closure.

**Methods:**

A BLAST search identified *LYM2* orthologs in the genomes of *Populus trichocarpa* and *Populus x canescens*. The poplar paralogs *PcLYM2–1* and *PcLYM2–2* were subsequently characterized on the molecular and functional level using chitin affinity purification and protein-tagging with fluorescent labels. The chitin response of knockout lines generated via the CRISPR/Cas9 approach was examined with particle bombardment assays.

**Results:**

*PcLYM2–2* exhibits tissue-specific alternative splicing, resulting in variants that differ exclusively at the first exon encoding the three LysM domains. Chitin-binding assays showed that all identified PcLYM2 proteins bind chitin. Subcellular localization studies in *Nicotiana benthamiana* indicated that all identified PcLYM2 proteins localize at plasmodesmata, suggesting a specific subcellular function of these proteins. Plasmodesmal flux analysis of wildtype poplar and *Pclym2–1 Pclym2–2* double knockout lines demonstrated that PcLYM2 proteins mediate chitin-triggered PD closure. Loss-of-function of *PcLYM2–1* is sufficient to abolish chitin-induced PD closure, suggesting that *PcLYM2-1* is an essential LysM-RLP involved in this process.

**Conclusion:**

Poplar regulates plasmodesmal flux after chitin detection through *LYM2* orthologs. The expression of *LYM2* variants may modify chitin perception and signaling in different tissues of poplar.

## 1 Introduction

During their life cycle, plants are exposed to abiotic and biotic stresses and the ability to perceive environmental signals is crucial for an adequate response ensuring plant survival. Non-pathogenic and pathogenic microbes are recognized by plants upon detection of microbe- or pathogen-associated molecular patterns (MAMPs or PAMPs) (Antolín-Llovera et al., 2014; Boutrot and Zipfel, 2017). These are ‘non-self’ molecules derived from microorganisms that generally have a conserved chemical structure within a group of microorganisms (Choi and Klessig, 2016; Yu et al., 2021), such as bacterial lipopolysaccharides, flagellin22 (flg22), elongation factor (EF-Tu), lipoproteins, peptidoglycans and fungal chitin. Plant innate immunity that is initiated upon the perception of PAMPs is termed PAMP-triggered immunity (PTI).

Microbial signals are perceived by pattern recognition receptors (PRRs) (Antolín-Llovera et al., 2014; Bender and Zipfel, 2023; Bernoux et al., 2022; Yu et al., 2021). Plant PRRs are surface-localized proteins such as receptor-like kinases (RLKs) or receptor-like proteins (RLPs) [4-6]. RLKs have an ectodomain for ligand binding, a transmembrane domain and an intracellular kinase domain for signal transduction. RLPs lack the intracellular kinase domain. Plant PRRs are able to activate one or more signaling pathways and often form a complex with co-receptors to initiate plant immune signaling (Steinbrenner, 2020; Bender and Zipfel, 2023).

Fungal pathogens can be recognized by plants through the perception of the PAMP chitin, the main component of fungal cell walls that is a homopolymer of β-1, 4-linked N-acetylglucosamine (GlcNAc) (Kaku et al., 2006), which can be recognized by LysM-containing receptors (Buist et al., 2008; Antolín-Llovera et al., 2014; Kaku and Shibuya, 2016). In the model plants *Arabidopsis thaliana* and *Oryza sativa*, the components of the chitin receptor complex have been well characterized. Chitin recognition in Arabidopsis is mediated by CHITIN ELICITOR RECEPTOR KINASE1 (CERK1), a plasma membrane-localized Lysin motif receptor-like kinase (LysM-RLK) that is considered to be an essential component of the Arabidopsis chitin receptor complex (Miya et al., 2007; Petutschnig et al., 2010). CERK1 associates with additional LysM-RLKs, LYK4 and LYK5, in order to facilitate proper chitin-induced signaling (Wan et al., 2012; Cao et al., 2014; Erwig et al., 2017; Xue et al., 2019; Mittendorf et al., 2024). Homologs of LysM-RLKs have been identified in the poplar genome (Cope et al., 2023).

In rice, the major component of the chitin receptor complex is CHITIN ELICITOR BINDING PROTEIN (CEBiP), a GPI-anchored LysM-RLP that does not have a kinase domain and requires association with OsCERK1 in order to trigger chitin-induced plant defenses (Kaku et al., 2006; Miya et al., 2007; Shimizu et al., 2010; Kouzai et al., 2014; Desaki et al., 2019). A “sandwich-type” rice receptor complex consisting of two CERK1-CEBiP heterodimers that bind chitin was proposed (Hayafune et al., 2014). OsCEBiP contains three extracellular LysM domains, and the central LysM domain is important for binding to chitin due to the presence of hydrophobic residues, that act as chitin-binding site (Hayafune et al., 2014; Liu et al., 2016). There are three orthologs of OsCEBiP in Arabidopsis, LYSIN MOTIF DOMAIN-CONTAINING GLYCOSYLPHOSPHATIDYL INOSITOL-ANCHORED PROTEIN (LYM) 1, LYM2, and LYM3. Only LYM2 was shown to bind chitin (Fliegmann et al., 2011; Shinya et al., 2012), while the other two homologs, LYM1 and LYM3, were reported to bind peptidoglycan (PGN) and mediate PGN-induced signaling in association with CERK1 (Willmann et al., 2011). The chitin binding homolog in Arabidopsis, LYM2, is not involved in CERK1-mediated chitin responses (Shinya et al., 2012), but is essential for chitin-induced suppression of plasmodesmal flux independently of CERK1 (Faulkner et al., 2013; Cheval et al., 2020).

Plasmodesmata (PD) are cytoplasmic channels that connect cytoplasm, endoplasmic reticulum (ER) and plasma membrane (PM) of adjacent cells, establishing a supracellular network within plant cells (Bayer and Benitez-Alfonso, 2024; Tee and Faulkner, 2024; Zanini and Burch-Smith, 2024). The continuity of the plasma membrane across plasmodesmata is called plasmodesmal plasma membrane (PD-PM) while the plasmodesmal element connecting the ER of adjacent cells is called desmotubule (Sager and Lee, 2018). The PD-PM contains lipid components that are highly different to those found in the bulk PM, e.g. an enrichment of sterols and sphingolipids with very long chain saturated fatty acids (Grison et al., 2015; Sager and Lee, 2018). Some glycosylphosphatidyl inositol (GPI)-anchored proteins show preferential association with the sterols and sphingolipids that are abundant at the PD-PM (Galian et al., 2012; Zavaliev et al., 2016). *Arabidopsis thaliana* LYM2 (AtLYM2) is a GPI-anchored protein that localizes to both PM and PD-PM. The perception of fungal chitin was shown to promote PD-closure in an AtLYM2 dependent manner (Faulkner et al., 2013; Cheval and Faulkner, 2018; Cheval et al., 2020).

Poplar (*Populus* spp.) is an established model system for studying tree species due to the available genetic information, a small genome size of around 500 Mbp, simplicity of genetic transformation and high capacity for *in vitro* regeneration (Bradshaw et al., 2000; Tuskan et al., 2006; Thakur et al., 2021; Lu et al., 2025). Poplar is widely used in the pulp and paper industry and considered as a potential bioenergy resource (Bryant et al., 2020; An et al., 2021). Woody perennials, like poplar, have extended life cycles, exhibit continuous secondary growth, and undergo recurring periods of dormancy. Trees must survive over much longer timescales than their pathogens, which often have shorter life cycles and therefore a greater capacity for rapid evolutionary adaptation that can overcome existing defense mechanisms (Polle et al., 2013). Thus, perennials may have defense systems that differ from annual plants because contrasting life histories expose annuals and perennials to fundamentally different pathogen pressures. Many poplar plantations are facing a severe problem due to infections caused by the rust fungus, *Melampsora larici-populina*, leading to huge losses of poplar biomass (Duplessis et al., 2009; Hacquard et al., 2011). The identification of putative *Melampsora* effectors that may affect plasmodesmata (Germain et al., 2018; Rahman et al., 2021) suggests that proper plasmodesmatal function plays a crucial role in limiting *Melampsora* proliferation.

This prompted us to identify LYM2 homologs in poplar and investigate whether they are involved in chitin-triggered defense signaling and plasmodesmal flux. Two LYM2 homologs were found in the genome of *Populus* x *canescens*. One of the paralogs exhibits tissue-specific, alternative splicing, resulting in two LYM2 variants, which differ in their LysM domains. Both poplar LYM2 paralogs, including the splicing variants, are chitin-binding proteins that reside in both PM and PD-PM, pointing to a role in controlling PD function after chitin perception. The poplar LYM2 proteins may form homo- and heterodimers and differential LYM2 protein complex formation may enable tissue-specific modulation of chitin perception and signaling.

## 2 Materials and methods

### 2.1 Plant materials and growth condition

The hybrid *P.* x *canescens* (*P. tremula* x *P. alba*) clone INRA 717-1B4 was used in this research. *In vitro* propagation of both wild type and transgenic plantlets was conducted on half-strength Murashige-Skoog (½ MS) medium (Duchefa Biochemie BV) supplemented with 2% (w/v) sucrose. Potting on soil was done on Fruhstorfer Erde Typ T25 (Hawita Gruppe GmbH) supplemented with 5% (v/v) washed sand. *In vitro* grown plantlets and potted plants were cultivated under long day conditions (light: 16h at 22 °C, dark: 8h at 18 °C, 60% relative humidity, light intensity at 70–80 μmol m^−2^ s^−1^).

### 2.2 Phylogenetic analysis of poplar LysM-RLPs

Protein sequences of putative LysM-RLPs were obtained by performing BLASTP searches at Phytozome (http://phytozome-next.jgi.doe.gov) *Populus trichocarpa* (*Pt*) Nisqually genome v3.0. The *A. thaliana* LYM1, LYM2 and LYM3 sequences were used as queries. For phylogenetic tree construction, the protein sequences of LysM-RLPs from rice were included. A multiple sequence alignment of the Arabidopsis, rice and identified poplar protein sequences using CLUSTALW (www.ebi.ac.uk/jdispatcher/msa/clustalo) was performed. A phylogenetic tree was constructed in MEGA11 (Molecular evolutionary genetics analysis version 11; (Tamura et al., 2021) based on the neighbor-joining method (1000 bootstrap replicates). Evolutionary distances were calculated using the Poisson correction method.

### 2.3 Protein domain analysis

The domain organization of putative LYM2 proteins was analysed by using the following prediction tools: The SignalP 4.1 server (Petersen et al., 2011) was used for assignment of signal peptides (SP), InterPro Scan integrated in Geneious^®^ 8.1.9 (Quevillon et al., 2005; Kearse et al., 2012) for LysM domain prediction, KohGPI (Fankhauser and Mäser, 2005) and PredGPI (Pierleoni et al., 2008) for identification of a putative omega site (ω) as GPI attachment signal.

### 2.4 cDNA cloning and sequence analyses

Primers for PCR amplification of genes encoding putative LysM-RLPs were designed using information available from the genome sequences of the *P. tremula* and *P. alba* haplotypes on Phytozome (https://phytozome-next.jgi.doe.gov/) (**Supplementary Table 2**). RT-PCR to amplify the candidate genes was conducted using phusion DNA polymerase (F-530L, Thermo Scientific) and cDNA from leaf tissue as a template. TA cloning (TA cloning KIT 45-0046, Invitrogen) of the purified amplicon was done to obtain allele-specific sequences of each gene. The clone-derived plasmids were sequenced (Microsynth Seqlab) and further analysed using Geneious 8.1.8 software (Biomatters Ltd). Predicted open reading frames (ORFs) of the obtained sequences were compared with the *PcLYM2* sequences of *P.* x *canescens* extracted from *P. tremula* x *alba* haplotype genomes. Verification of alternative splicing of *PcLYM2–2* was done on the transcriptomic and on the genomic level by amplifying and sequencing the full length cDNAs and genomic sequences from *P.* x *canescens*.

### 2.5 RNA extraction and expression analyses

RNA was isolated from five different tissues (roots, wood, developing xylem, bark and leaves) of soil-grown *P.* x *canescens* that were cultivated either in a long day climate chamber or in the greenhouse. The harvested plant materials were immediately frozen in liquid nitrogen before being ground and used for RNA isolation using the CTAB extraction protocol according to Chang et al. (1993). The extracted RNA was reverse transcribed into cDNA (cDNA synthesis Kit #K1631, Thermo Scientific), and used as a template for qPCR to measure the transcript abundance of genes encoding PcLYM2, using primers listed in the **Supplementary Table 2**. qPCR was performed using the SsoFast EvaGreen Supermix (Bio-Rad) and ubiquitin was used as a reference to calculate the relative expression levels. The efficiencies of primers used for qPCR were checked on gDNA, showing an amplification efficiency of 89-95%.

### 2.6 Chitin-affinity purification of poplar leaf proteins

Chitin beads (New England Biolabs) where washed three times with ddH_2_O. 10g of leaf material of soil grown plants was ground in liquid nitrogen and 100 ml extraction buffer (250 mM sucrose, 5% (v/v) glycerol, 1 mM Na_2_MoO_4_ x 2 H_2_O, 25 mM NaF, 10 mM EDTA, 1 mM DTT, 0.5% (w/v) Triton X-100, 100 mM HEPES-KOH pH 7, 5) with protease inhibitors (0.20 mM AEBSF, 0.72 µM bestatin hydrochloride, 0.72 µM pepstatin A, 10 µM leupeptin hemisulfate, 1.4 µM E-64, 2.53 mM phenanthroline) and 0.5% PVPP was added. Tubes were centrifuged 10 min at 4 °C, 1000 rpm and the supernatant was filtered through 50 µm CellTrics^®^ filters (Sysmex Deutschland GmbH). Chitin magnetic beads were added and extracts were incubated for one hour at 4 °C. Chitin beads with bound proteins were separated from the extract with a magnetic rack. The chitin beads were washed two times with TBS-T buffer (3 M NaCl, 1% (v/v) Tween-20, 200 mM TRIS, pH 8.0), once with TBS-T buffer containing 500 mM NaCl and once with TBS-T buffer without NaCl. The chitin beads were taken up in 4x SDS buffer (400 mM DTT, 8% (w/v) SDS, 40% (v/v) glycerol, 0.1% (w/v) bromphenol blue). Proteins bound to the magnetic beads were eluted at 95 °C. The chitin pull down was processed via SDS gel electrophoresis for mass spectrometry analyses.

### 2.7 Tryptic digestion of proteins and purification for LC-MS/MS analyses

Proteins in the molecular weight range of interest were cut from SDS-PAGE gels. Gel slices were washed three times with water. Subsequently, the water was exchanged with acetonitrile and samples were incubated for 10 min. After removal of acetonitrile, the gel slices were dried in a SpeedVac (Eppendorf) at 45 °C. Samples were incubated for one hour at 56 °C in 10 mM DTT, 100 mM NH_4_HCO_3_. The DTT solution was exchanged with 55 mM iodoacetamide in 100 mM NH_4_HCO_3_ for alkylation of the reduced cysteine residues and samples were incubated for 45 min in the dark. Gel slices were washed in 100 mM NH_4_HCO_3_ for 15 min. A second washing step was performed with acetonitrile for 15 min. The acetonitrile was removed and the gel slices dried in a SpeedVac for 10 min at 45 °C. Trypsin digestion was performed overnight at 37 °C. The next day, gel slices were incubated in 20 mM NH_4_HCO_3_ and twice in 50% acetonitrile, 5% formic acid for 10 and 20 min, respectively. Supernatants were collected and dried in a SpeedVac at 45 °C and resuspended in proteomics sample buffer (2% acetonitrile, 0.1% formic acid). Protein samples were purified for MS analyses with a C18 stage tip (CDS Empore™, Thermo Fisher Scientific). Eluted peptide samples were dried using a SpeedVac at 45 °C. The dry pellet was suspended in 20 µl proteomics sample buffer (2% acetonitrile, 0.1% formic acid) and submitted for LC/MS analysis.

### 2.8 LC-MS/MS analysis

LC/MS-MS analyses were performed with an RSLCnano Ultimate 3000 chromatography system coupled to an Orbitrap Velos Pro mass spectrometer (both Thermo Fisher Scientific). Peptides were separated by reverse phase chromatography on an Acclaim PepMap RSLC column (Thermo Fisher Scientific) with a water-acetonitrile gradient. Eluted peptides were online ionized by nano-electrospray at 2.3 kV using the Nanospray Flex Ion Source (Thermo Fisher Scientific). Full scans of the ionized peptides were recorded in the range of 300–1850 m/z within the Orbitrap-FT analyzer at a resolution of 30000. In parallel, data-dependent top 15 fragmentation spectra were acquired by collision-induced dissociation (CID) in the LTQ Velos Pro linear ion trap. The XCalibur 2.2 software (Thermo Fisher Scientific) was used for LC/MS method programming and data acquisition. Protein database searches and subsequent data analyses were carried out using MaxQuant (V.1.6.10.43; (Cox and Mann, 2008) using the default parameters and the haplotype-specific protein databases PtremulaxPopulusalbaHAP1_717_v5.1.protein.fa and PtremulaxPopulusalbaHAP2_716_v5.1.protein.fa (Phytozome 13, http://phytozome-next.jgi.doe.gov). The mass spectrometry proteomics data have been deposited to the ProteomeXchange Consortium (Deutsch et al., 2023) via the PRIDE (Perez-Riverol et al., 2022) partner repository with the dataset identifier PXD058986.

### 2.9 Cloning of CRISPR/Cas9 constructs

Single guide RNA (sgRNA) expression cassettes carrying the target sequences were generated according to the required parameters described in Ma et al. (2015; 2016). In total, four sgRNAs were designed using primers listed in the **Supplementary Table 2** to simultaneously modify target sites (T1-T4) located after the LysM domains and before the omega (ω) site of all PcLYM2 homologs (both alleles including the splicing variants) in order to disrupt the function of the omega (ω) site, which is crucial for the attachment of the GPI anchor to the proteins. The promoters of each expression cassette were amplified from intermediate vectors (AtU3d, AtU3b, AtU6–1 and AtU6-29) obtained from Addgene. These multiple sgRNA expression cassettes encoding the sgRNAs, AtU3d-sgRNA1_AtU3b-sgRNA2_AtU6-1-sgRNA3_AtU6-29-sgRNA4 in this respective order, were then cloned into pYLCRISPR/Cas9P_35S_-N (Addgene) through Gibson assembly reactions (Gibson et al., 2009) at 50 °C for 1h (NEBuilder, New England Biolabs), using primers listed in the **Supplementary Table 2**. The assembled product was transformed into *E. coli* DH5-α competent cells and transformants were grown overnight at 37 °C. Plasmids isolated from the positively selected clones were sequenced to check for the proper inserts and further transformed into *Agrobacterium tumefaciens.*

### 2.10 Poplar transformation

*Agrobacterium*-mediated plant transformation was carried out according to a protocol adopted from Matthias Fladung, Thünen Institute of Forest Genetics, Großhansdorf, Germany. Stems of *in vitro* cultivated *P.* x *canescens* were cut into segments of about 3–8 mm, and incubated in an *Agrobacterium* suspension (OD_600_ = 0.25-0.8) supplemented with 20 µM acetosyringone (Sigma-Aldrich) for 30 minutes at 28 °C, 120 rpm shaking in the dark. Stems were transferred onto co-incubation medium (½ MS supplemented with 2% sucrose, solidified with 0.7% agar, pH 5.8) and incubated in the dark at 22 °C for 2–3 days. Subsequently, the explants were washed several times using sterile ddH_2_O supplemented with 400 µg/ml timentin before transfer onto selection medium (½ MS supplemented with 2% sucrose, 0.01% Pluronic, 0.01 µM Thidiazuron, 50 mg/l cefotaxime, 200 mg/l timentin and 50 mg/l kanamycin, solidified with 0.7% agar, pH 5.8). After 2–4 weeks regenerates were transferred onto ½ MS media supplemented with 2% (w/v) sucrose, 150 mg/l cefotaxime, 200 mg/l timentin and 50 mg/l kanamycin for rooting. Leaf samples of the rooting plants were collected to isolate the gDNA to examine editing of the target site using primers listed in **Supplementary Table 2**.

### 2.11 Transient gene expression in *Nicotiana benthamiana*

Coding sequences of LysM-RLPs (PcLYM2-1, PcLYM2-2.1 and PcLYM2-2.2) were amplified from cDNA of *P.* x *canescens* (primers listed in the **Supplementary Table 2**). Constructs for expression of *PcLYM2* genes in fusion with the mVenus fluorescent tag under control of the 35S promoter were generated by assembling the 35S fragment and the respective coding sequence of PcLYM2 paralogs and splicing variants as well as the mVenus coding sequence into the pGreenII-0229 vector (Hellens et al., 2000) via Gibson assembly (NEBuilder, New England Biolabs; Gibson et al., 2009). The generated constructs were transiently expressed in *Nicotiana benthamiana* leaves after leaf infiltration using a syringe. The transient assays were used for subcellular localization studies and chitin-binding assays.

### 2.12 Confocal microscopy

Microscopy analysis was conducted with the confocal laser scanning microscope (CLSM) Leica TCS SP5 system (Leica Microsystems) equipped with an argon laser. mVenus was excited at 514 nm and the emitted light was captured in the range of 530–569 nm. For plasmodesmal flux analysis, the eGFP cytoplasmic marker and mKate nuclear marker were excited at 488 nm and 561 nm, respectively, and the emitted lights were collected at a range of 500–540 nm for eGFP and 620–640 nm for mKate. Images shown in this work are maximum projections of 10 Z-stack series taken ~1 µm apart under an 40x objective for imaging subcellular localization of PcLYM2 proteins and under an 20x objective for plasmodesmal flux analysis. Images shown for plasmodesmal flux analyses are overlays of the eGFP and mKate chanel. Brightness and black level of images were adjusted. Adjustments were applied to the entire image.

### 2.13 Chitin-binding assay

Chitin-binding affinity assay was performed by isolation of proteins from *N. benthamiana* leaves transiently expressing mVenus fusions of PcLYM2 proteins. Total protein extract was collected by grinding 100 mg leaf materials in 1 ml protein extraction buffer (250 mM sucrose, 5% glycerol, 1 mM Na_2_MoO_4_ + 2H_2_O, 25 mM NaF, 10 mM EDTA, 1mM DTT, 0.5% Triton X-100, 100 mM HEPES-KOH pH 7.5) supplemented with protease inhibitors. For chitin-binding assays, total protein extracts of 500-1000 µg were incubated with 25 µl chitin magnetic beads (New England BioLabs) at 4 °C for 1h to isolate chitin-binding proteins. The chitin magnetic beads-bound protein was separated from the supernatant using a magnet, subsequently washed with pre-cooled 1x TBS-T (0.05% Tween-20, 10 mM Tris-HCl, pH 8.0) containing 150 mM NaCl followed by washing with water. Water from the last washing step was removed by pelleting the chitin magnetic beads-bound protein using a magnet, and the beads were mixed with SDS-loading dye. Western blot analysis was carried out to detect mVenus-tagged PcLYM2 in the total protein extract and in the protein fraction, which exhibits chitin binding, using an α-GFP antibody (Chromotek). Quantification of western blot signals was performed in Bio-Rad Image Lab 5.2.1 software by manual lane and band detection. Signals from chitin binding proteins were normalized to total protein extract signals.

### 2.14 Microprojectile/particle bombardment assay for plasmodesmal flux analysis

A plasmid encoding a mobile eGFP cytoplasmic marker and immobile mKate nuclear marker was used for particle bombardment with a Biolistic^®^ PDS-1000/He particle delivery system from Bio-Rad. 1.0 µm gold microcarriers (Bio-Rad) were coated with the plasmid DNA based on a modified protocol from Robert Hänsch, Department of Plant Biology, Technische Universität Braunschweig, Germany. For coating, 3 mg gold particles were pre-washed with 100 µl of 70% EtOH and vortexed for 20 s. Centrifugation at 6000 rpm for 1 min was performed to collect the gold particles. The gold microcarriers were washed with 50 µl ddH_2_O, and centrifugation at 2000 rpm for 1 min was done to remove the supernatant. 50 µl of pre-cooled 50% glycerin was added and the solution was vortexed for 20 s before incubation in an ultrasonic water bath for 10 s. A maximum of 15 µg plasmid DNA was added to the solution that was immediately pipetted up and down before being incubated on ice for 15 min. The suspension was added dropwise to 50 µl 2.5 M CaCl_2_ and then to 20 µl of 0.1 M spermidine (Sigma-Aldrich), and incubated on ice for 10 min. The pellets of DNA-coated gold particles were subsequently washed two times with 100 µl of 70% EtOH and 100 µl of 96-100% EtOH. The DNA-coated gold particles were added to 50 µl of 96-100% EtOH. For bombardment using a hepta-adapter, 7 µl of DNA-coated particles were loaded evenly on each macrocarrier (Bio-Rad). The third leaves of *in vitro* grown wild type *P.* x *canescens* as well as *PcLYM2* knock-out lines were detached from the plants and placed on a wet filter paper in a petri dish. The petri dish was positioned at a distance of 11 cm from the hepta-adapter and bombarded with DNA-coated gold particle using 900 psi rupture disks (#1652328 from Bio-Rad) that resulted in a final bombardment pressure of ~650–700 psi. The detached leaves were further infiltrated with 500 µg/ml chitin (C9752-5G from Sigma-Aldrich) or ddH_2_O 2h post bombardment and further placed on a wet filter paper in a petri dish, which was then closed with parafilm and cultivated under long day conditions. Imaging was done 48h post bombardment. The number of directly adjacent and surrounding cells showing eGFP signal was counted (Faulkner et al., 2013). Signals from bombarded cells in immediate vicinity to each other were excluded to avoid double counting of signals. Counting of cells showing eGFP signal was done three times by different persons in a blind study and the same differential responses of WT and knockout lines were obtained in all analyses.

### 2.15 ROS burst assay

Reactive oxygen species (ROS) burst was measured according to (Petutschnig et al., 2010). Leaf discs with a diameter of 4 mm were incubated overnight in 100 µl normal tap water using 96-well plates. The following day, water from each well was replaced with 100 µl solution containing 100 µM L-012 (120-04891, Wako Chemicals) and 10 µg/ml horseradish peroxidase (Sigma-Aldrich). For analysis of chitin-elicited ROS burst, 100 µg/ml chitin from shrimp shells (C9752-5G from Sigma-Aldrich) was added to the wells. The kinetic luminescence was detected every minute using Infinite M200 Tecan plate reader for 1 hour.

### 2.16 MAP kinase assay

The leaves of *in vitro* cultivated wild type *P.* x *canescens* as well as *PcLYM2* knock-out mutants (*Pclym2–1* and *Pclym2–1 Pclym2-2*) were cut at the petiole and then incubated in normal tap water overnight to allow the decline of wounding effects. The following day, leaves were vacuum infiltrated with either 10 µg/ml chitin (C9752-5G from Sigma-Aldrich), or water and harvested after 10 minutes incubation. Total protein extraction was carried out based on the protocol described in Petutschnig et al. (2010). Frozen leaf materials were ground and 500 µL extraction buffer (250 mM sucrose, 5% glycerol, 1 mM Na_2_MoO_4_ + 2H_2_O, 25 mM NaF, 10 mM EDTA, 1mM DTT, 0.5% Triton X-100, 100 mM HEPES-KOH pH 7.5) supplemented with protease inhibitors was added and the suspension was mixed thoroughly. Following 10 minutes centrifugation at 13000 rpm, the supernatant containing the total protein extract was collected. Protein concentrations were equalized and the same amount of total protein for each sample was mixed with SDS loading buffer for SDS-PAGE. Western blotting was carried out on SDS-PAGE using an antibody against phospho-p44/42 (Cell Signaling Technology). Relative signal intensity was analysed by densitometry (Image J, 1.54p, plugin “analyse gels”). Chitin induced signals were normalized to loading control).

### 2.17 Statistical analysis

Statistical analysis for chitin-binding was done by performing One-way ANOVA followed by Tukey’s test. qPCR data were analysed with Student’s t-test or One-way ANOVA followed by Tukey’s test. All data sets passed the normality test (Shapiro-Wilk) and data analysed with One-way ANOVA showed equal variance. For bombardment experiments, statistical analyses of counts of cells showing an eGFP signal was conducted in R version 4.4.1 (R Core Team 2024). A generalized linear model using a negative binomial distribution was fitted to the data using the function ‘glm’ from the package ‘stats’ (R Core Team 2024), applying the lines and the chitin treatment as factors. An Analysis of Deviance (ANODE) was applied to the model to test for significant interaction effects using the function ‘ANOVA’ from the ‘car’ package (Fox and Weisberg, 2019). Subsequently, a *post-hoc* test was conducted to determine homogeneous subsets, applying Tukey’s honest significance test using the function ‘glht’ from the package ‘multcomp’ (Hothorn et al., 2008).

## 3 Results

### 3.1 Identification of genes encoding putative LysM-RLPs in poplar

**Figure 1:**
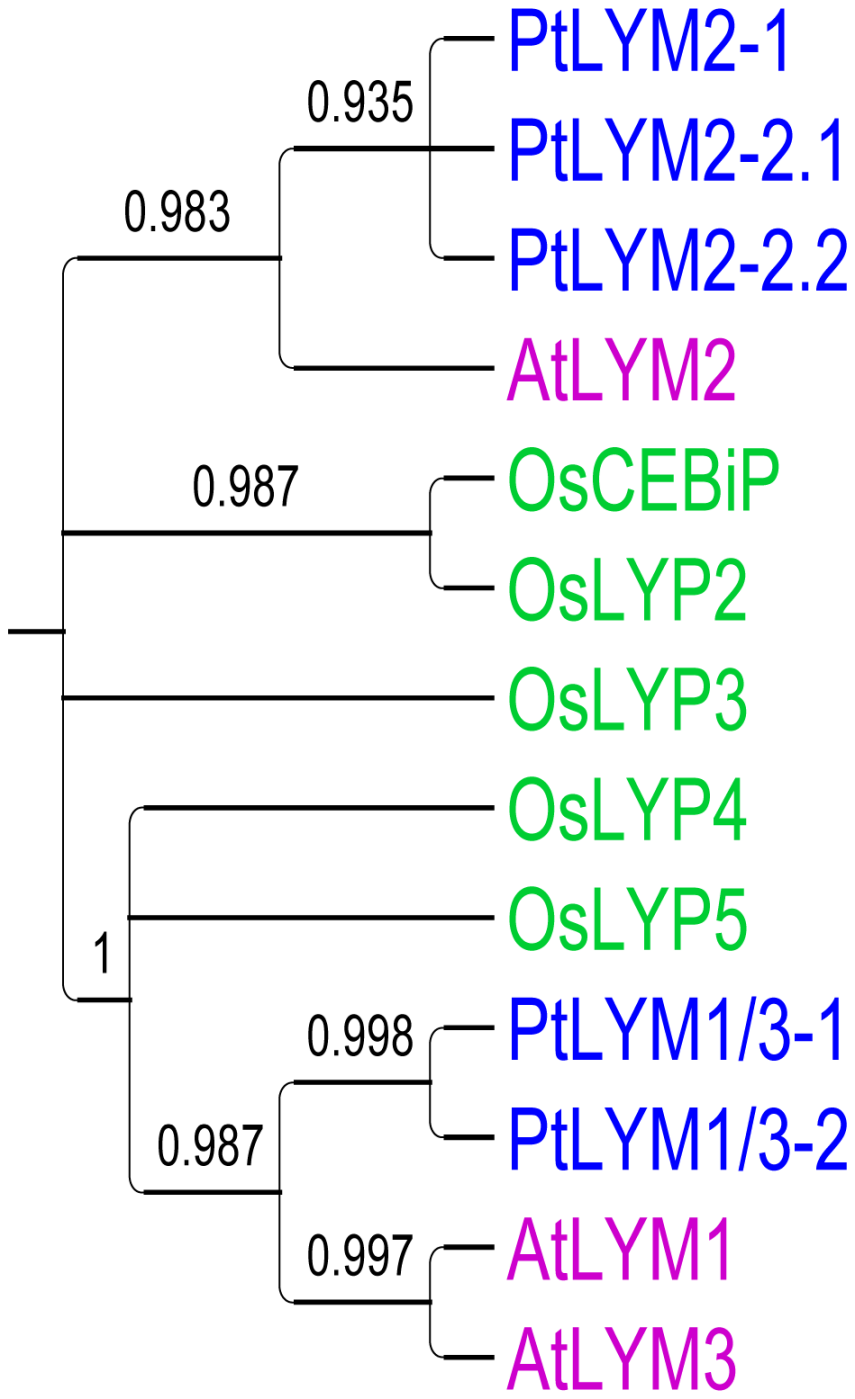
Two poplar LYM2 paralogs cluster with AtLYM2. A phylogenetic tree of LysM-RLPs was constructed based on protein sequence similarity of putative *P. trichocarpa* Nisqually LysM-RLPs (blue) with well-characterized LYM proteins from *Arabidopsis thaliana* (magenta) and *Oryza sativa* (green). Two poplar paralogs of AtLYM2, a GPI-anchored protein that is involved in chitin-binding in *A. thaliana*, were assigned to a cluster with the Arabidopsis protein AtLYM2. Note, that two splice variants are predicted for PtLYM2-2. Numbers on the clade are branch support values (%) from bootstrapping of 1000 replicates (analyzed with MEGA for maximum likelihood).

A BLASTP search was carried out in the *Populus trichocarpa* (*Pt*) Nisqually genome v3.0 using Phytozome (http://phytozome-next.jgi.doe.gov), to search for putative LysM-RLPs in poplar using the protein sequences of LysM-RLPs from *Arabidopsis thaliana* (AtLYM1, AtLYM2 and AtLYM3) as queries. Protein sequences from *Populus trichocarpa* Nisqually that showed the highest homology with the queries were extracted from the database and used for phylogenetic analysis. A phylogenetic tree was constructed based on protein sequence similarity of putative LysM-RLPs from *P. trichocarpa* Nisqually with LysM-RLP sequences from the model plants *Arabidopsis thaliana* and *Oryza sativa* (**Figure 1**). Two orthologs of LysM-RLPs, PtLYM2-1 (Potri.004G183500) and PtLYM2-2 (Potri.009G143300), were assigned to a cluster with AtLYM2 (**Figure 1**). The annotation of the poplar genome (*P. trichocarpa* v3.0) indicates that the second homolog, *PtLYM2-2*, has two splicing variants designated as Potri.009G143300.1 and Potri.009G143300.2 resulting in two different protein versions, PtLYM2-2.1 and PtLYM2-2.2. In addition, two putative LysM-RLPs that are closely related to AtLYM1 and AtLYM3, LysM-RLPs involved in bacterial peptidoglycan perception (Willmann et al., 2011), were also identified in poplar (**Figure 1**).

**Figure 2:**
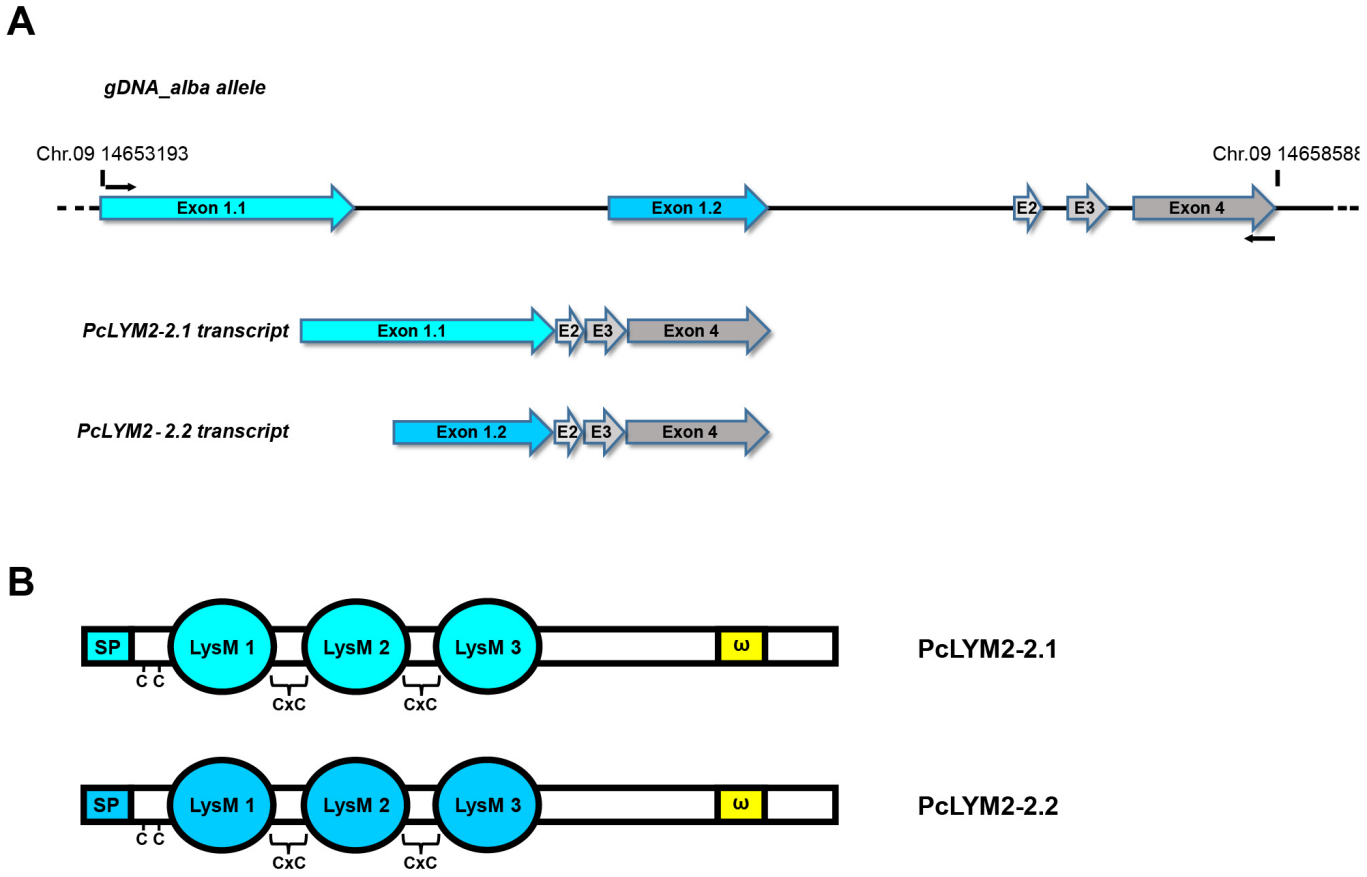
*PcLYM2–2* transcription and processing generate two splicing variants with alternative first exons encoding the LysM-domains. **(A)** Genomic arrangement of *PcLYM2–2* mapped to the scaffold of *P.* x *canescens* from the *PtremulaxPopulusalba*HAP2_716_v5.1 database (Phytozome 13, http://phytozome-next.jgi.doe.gov), illustrating the splicing event of this paralog that results in distinct first exons (highlighted in turquoise for *PcLYM2-2.1* and blue for *PcLYM2-2.2*), giving different transcript variants. Arrows indicate the primer positions for amplification of the sequence from gDNA or cDNA. Different lengths of exon 1.1 and exon 1.2 are due to different lengths of the 5’ UTRs of the respective transcripts (Potri009G143300.1 and Potri009G143300.2. **(B)** LYM2-2.1 and LYM2-2.2 protein domains. LysM-domains separated by CxC motifs are shown in turquoise and blue, respectively. The omega site for GPI attachment after processing of the C-terminus is indicated in yellow. SP, signal peptide; ω, omega site.

**Figure 3:**
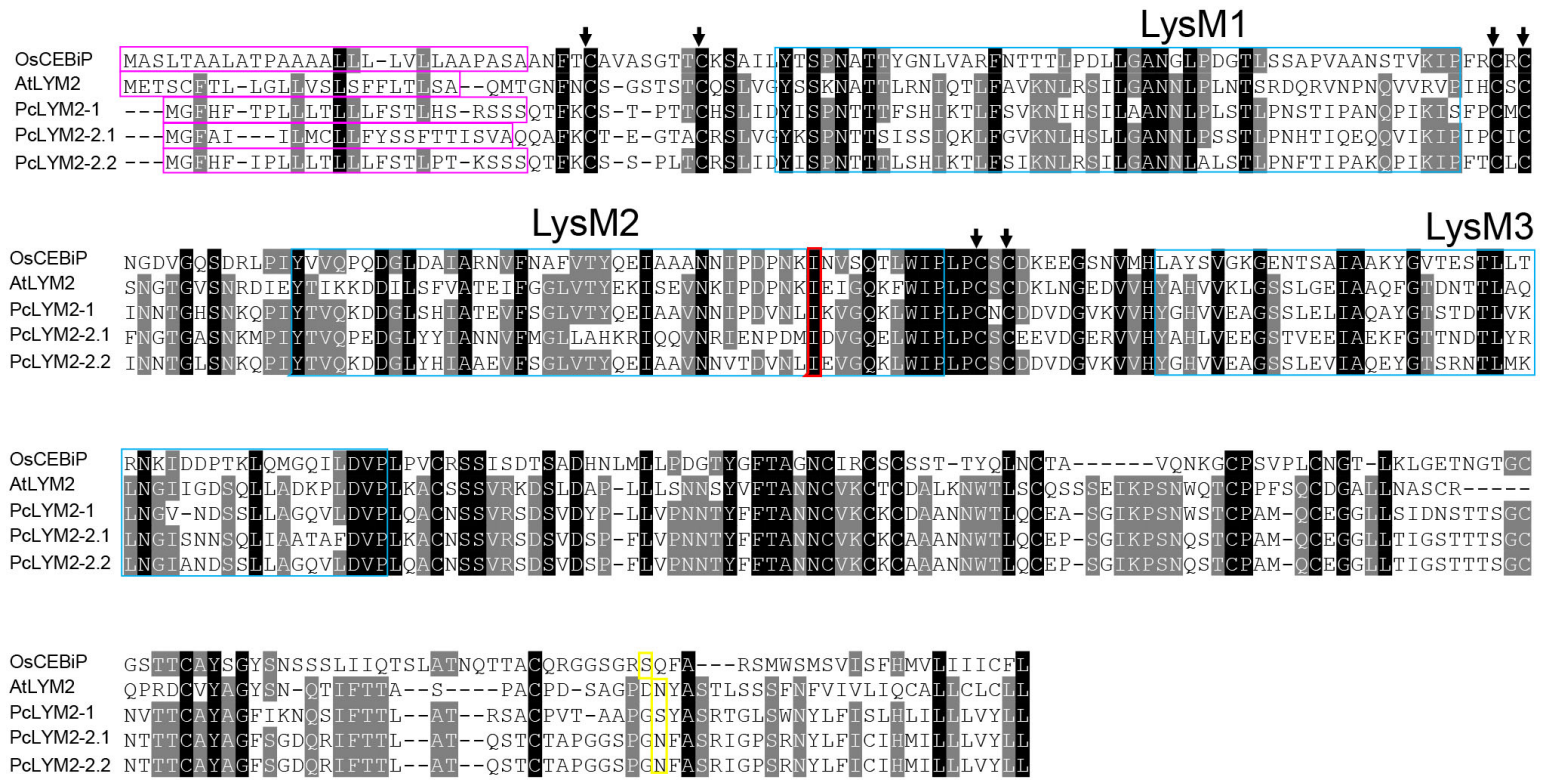
Alignment of putative LYM2 proteins from *Populus* x *canescens* with rice CEBiP and Arabidopsis LYM2 shows the conserved domain structure of the poplar LYM2 candidates. The amino acids encoding the signal peptide are indicated in magenta, LysM-domains in light blue and the GPI anchor omega sites in yellow. Conserved cysteine residues at the N-terminus and CxC motifs between LysM domains are indicated by arrows. The conserved isoleucine122 (numbering refers to the mature rice CEBiP protein without signal peptide) that is essential for chitin-binding is highlighted in red. Proteins encoded by the *alba* alleles were used for the alignment.

For further analysis, we used the *Populus* x *canescens* INRA 717-1B4 clone. *Populus* x *canescens* is a hybrid of *P. tremula* and *P. alba* with a genome containing alleles of both genotypes. Sequences of the putative *LysM-RLP* genes that are annotated in the *P. trichocarpa* Nisqually genome were identified in the *Populus tremula x Populus alba HAP1/HAP2 v5.1–tremula/alba* haplotypes (https://phytozome-next.jgi.doe.gov/) and termed *PcLYM2–1* and *PcLYM2-2.* Primers were designed for full-length cDNA amplification of the two paralogs as well as the predicted splicing variants from *P.* x *canescens* (*Populus tremula* x *alba* INRA 717-1B4) using leaf-derived cDNA. The *PcLYM2* amplicons were cloned and sequenced. Alleles of the *tremula* and *alba* haplotypes were identified. The sequencing of cDNAs revealed that the predicted splicing variants of *PcLYM2-2*, designated as *PcLYM2-2.1* and *PcLYM2-2.2*, are present on the mRNA-level in *P.* x *canescens*. The corresponding exon-intron structure of the *PcLYM2-2* gene was verified by amplifying its full genomic DNA sequence (**Figure 2A**). Mapping of the obtained *PcLYM2–2* cDNAs to the *PcLYM2–2* genomic sequence indicated that mutually exclusive alternative splicing occurs (Zhang et al., 2015; Chaudhary et al., 2019), giving a different exon 1 for each variant (**Figure 2A**). Exon 1 encodes three LysM domains, thus the two protein variants harbour different versions of LysM 1, 2 and 3 (**Figures 2B**, **3**). In both proteins, the extracellular LysM domains are separated by CxC motifs. Exon 2, 3 and 4, which encode the C-terminal part of the protein including the GPI-anchor site, are the same in both *PcLYM2-2.1 and PcLYM2-2.2* (**Figure 2B**).

PcLYM2–1 is highly similar to PcLYM2-2.2 with sequence identity of 86.8%, and PcLYM2-2.1 is the least similar one with sequence identity of 57.9%. The predicted protein sequences were also aligned to the well-characterized LysM-RLPs in rice and Arabidopsis, OsCEBiP and AtLYM2, respectively, to provide additional *in silico* support for the predicted protein domains and the presence of conserved amino acid residues, in particular isoleucine122/150 (numbering refers to OsCEBIP without and with signal peptide), that is essential for chitin-binding (Hayafune et al., 2014; Liu et al., 2016) (**Figure 3**).

Chitin-binding by PcLYM2 proteins enables their capture via chitin-coated magnetic beads. Mass spectrometry analysis of chitin pull-downs from leaf protein extracts was conducted to determine whether the splice variant–encoded PcLYM2–2 proteins are present in the *P. × canescens* proteome, provided they are chitin-binding.

**Figure 4:**
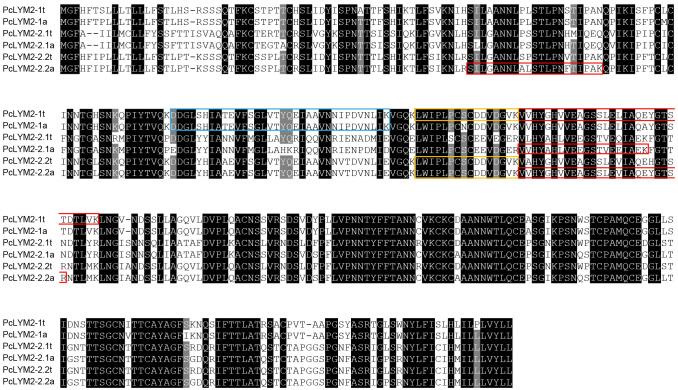
LYM2 proteins were identified in leaves of *Populus* x *canescens*. A mass spectrometry analysis was performed with proteins from leaf samples by chitin pull-down. Detected peptides are boxed. Peptides marked with a red box uniquely identify an ortholog (*tremula*, *alba*) of one protein (LYM2-1, LYM2-2.1, LYM2-2.2). Peptides boxed in turquoise identify a protein but do not discriminate between orthologs. Peptides shared between two or more LYM2 proteins are indicated with a yellow box. Proteins with the extension “t” and “a” are encoded by the *tremula* allele and *alba* allele, respectively.

The analysis showed that PcLYM2-1 and both PcLYM2–2 proteins are synthesized in *P.* x *canescens* and all three proteins bind chitin (**Figure 4**). Unique peptides (marked in red in **Figure 4**) of the PcLYM proteins could be assigned explicitly either to the *tremula* or the *alba* allele (labelled as “t” and “a” in **Figure 4**).

**Figure 5:**
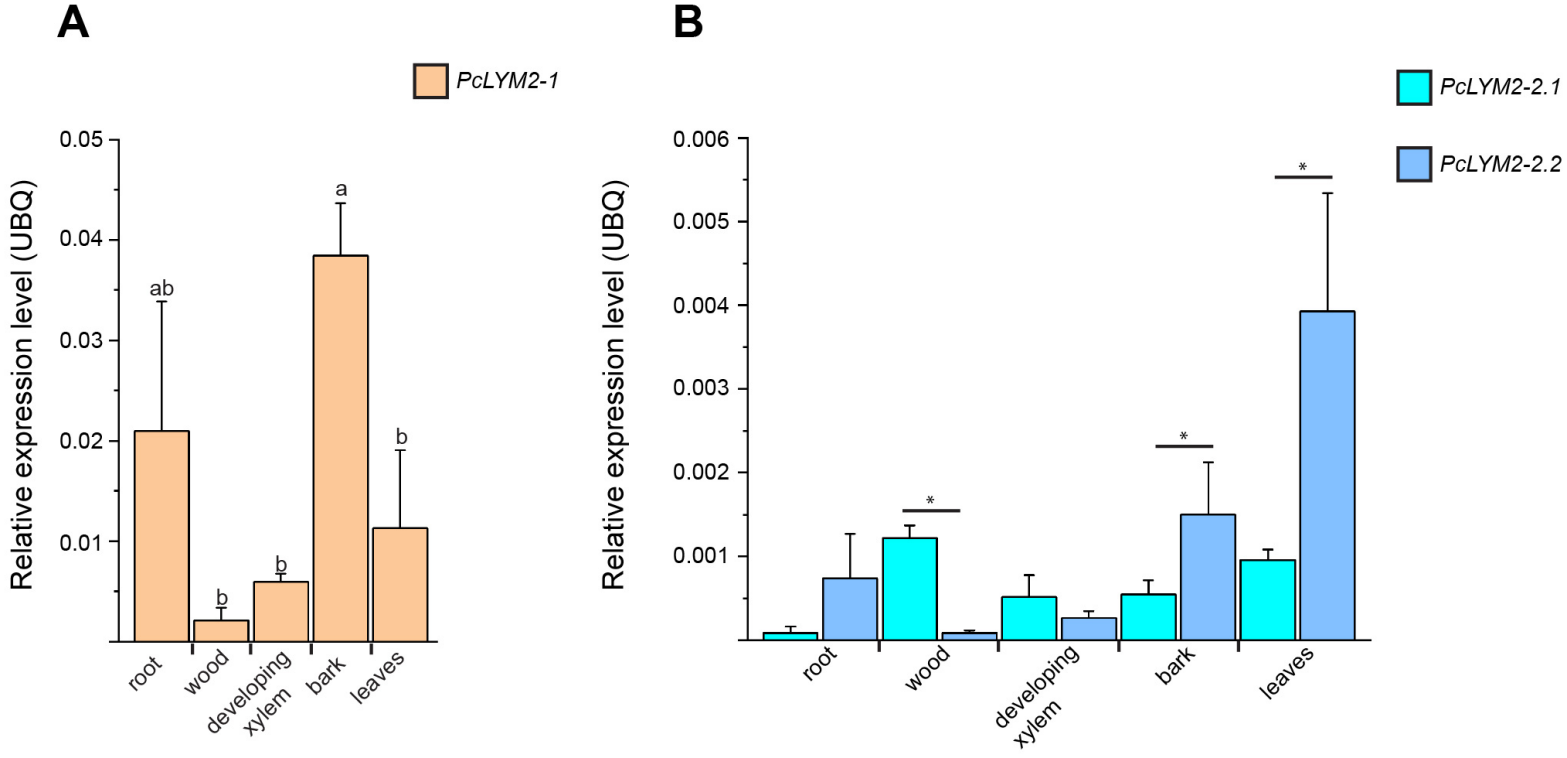
*PcLYM2* paralogs show tissue-specific expression levels. cDNA synthesized from RNA from different tissues of *Populus* x *canescens* was used as a template to perform qRT-PCR. Universal primers were selected to amplify cDNA of both the *alba* and the *tremula* allele. The transcript abundance was determined relative to UBQ as a reference. **(A)** Expression level of *PcLYM2-1*. Statistical analysis was conducted by one-way ANOVA followed by Tukey’s posthoc test. Different letters indicate statistically significant differences (*P* ≤ 0.05). Differences in expression values between bark-wood, bark-developing xylem and bark-leaves are statistically significant with P values of 0.0007, 0.002 and 0.007. **(B)** Expression level of *PcLYM2–2* splicing variants. Statistical analysis was performed with unpaired student’s t-test (*P* ≤ 0.05), statistical significancy is designated as *. Differences in transcript abundances between *PcLYM2–2* splicing variants in wood, bark and leaves are statistically significant with P values of 0.0008, 0.0043 and 0.0344. Data are means + SD (n = 3 biological replicates).

RT-PCR (qRT-PCR) expression analyses in root, wood, developing xylem, bark and leaves indicated that all *LYM2* genes are expressed throughout the plant (**Figure 5**). *PcLYM2–1* showed consistently higher expression than the *PcLYM2–2* variants across all examined tissues, with two exceptions. In wood, the expression levels of *PcLYM2–1* and *PcLYM2-2.1* were comparable and fell within the same order of magnitude. In leaves, transcript abundances of *PcLYM2–1* and *PcLYM2-2.2* are similar (**Figures 5A, B**). With *PcLYM2-2*, the splicing variant *1* exhibited higher expression in wood (P = 0.0008), while variant 2 showed significantly higher transcript levels in the bark (P = 0.0043) and leaves (P = 0.0344) (**Figure 5b**), suggesting distinctive functions of the splicing variants.

### 3.2 PcLYM2 proteins localize at PM and PD-PM

**Figure 6:**
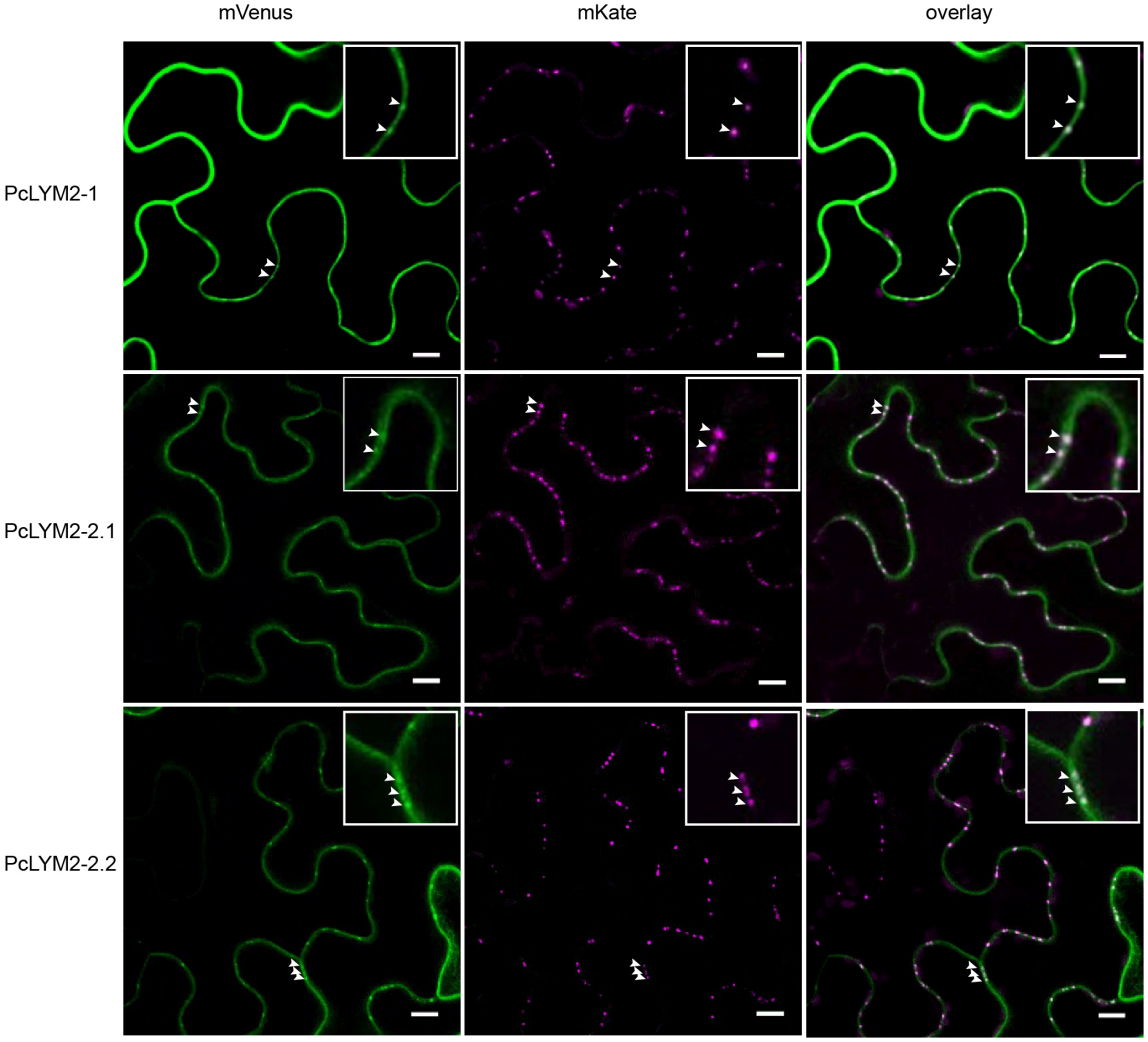
PcLYM2 proteins reside at both the plasma membrane and plasmodesmal plasma membrane. *Agrobacteria* strains for expression of *PcLYM2* in fusion with *mVenus* were co-infiltrated with an *Agrobacterium* strain containing a *PDLP5-mKate* construct as a marker for PD-localization into *N. benthamiana* leaves. Fluorescent marker localization was observed 72 hours post infiltration under a CLSM SP5, and the displayed images are maximum projections of 10 Z-stack series taken 1 µm apart with a 40x objective. In the left panel, images from the mVenus channel show the plasma membrane (PM) and plasmodesmal plasmamembrane (PD-PM) localization of PcLYM2 proteins. The central panel displays images of PDLP5-mKate as a marker for PD-localization visible as magenta dots. In the right panel, overlay images of mVenus and mKate channels confirm that PcLYM2 proteins co-localize with the PD-marker PDLP5 in PD-PM (exemplarily shown by arrow heads). Scale bar = 10 µm.

Subcellular localization of PcLYM2 proteins was analysed in leaves of *N. benthamiana* by *Agrobacterium*-mediated transient expression of *P_35S_:SP-mVenus-PcLYM2-1*, *P_35S_:SP-mVenus-PcLYM2-2.1* and *P_35S_:SP-mVenus-PcLYM2-2.2* (SP: signal peptide of the corresponding protein). CLSM analysis showed that PcLYM2-mVenus signals were unevenly distributed along the plasma membrane (PM) (**Figure 6**, left panel) giving a first hint that PcLYM2 proteins are enriched at the plasmodesmata. To demonstrate that PcLYM2 are indeed components of plasmodesmata, *PcLYM2* constructs were co-infiltrated with a plasmid expressing *PDLP5*, a PD-localized protein (Lee et al., 2011), tagged with mKate as fluorescent marker (**Figure 6**, middle and right panel). The dot-like PcLYM2-mVenus signals co-localized with the PDLP5-mKate fluorescence, thus confirming the PD localization. In contrast, the plasma membrane localized protein AtLYK5-Venus (Erwig et al., 2017; Cheval et al., 2020) did not show punctate fluorescence signals (**Supplementary Figure 1**). In conclusion, the data suggest that all identified PcLYM2 proteins localize at both PM and PD-PM.

### 3.3 PcLYM2 proteins are chitin-binding proteins

**Figure 7:**
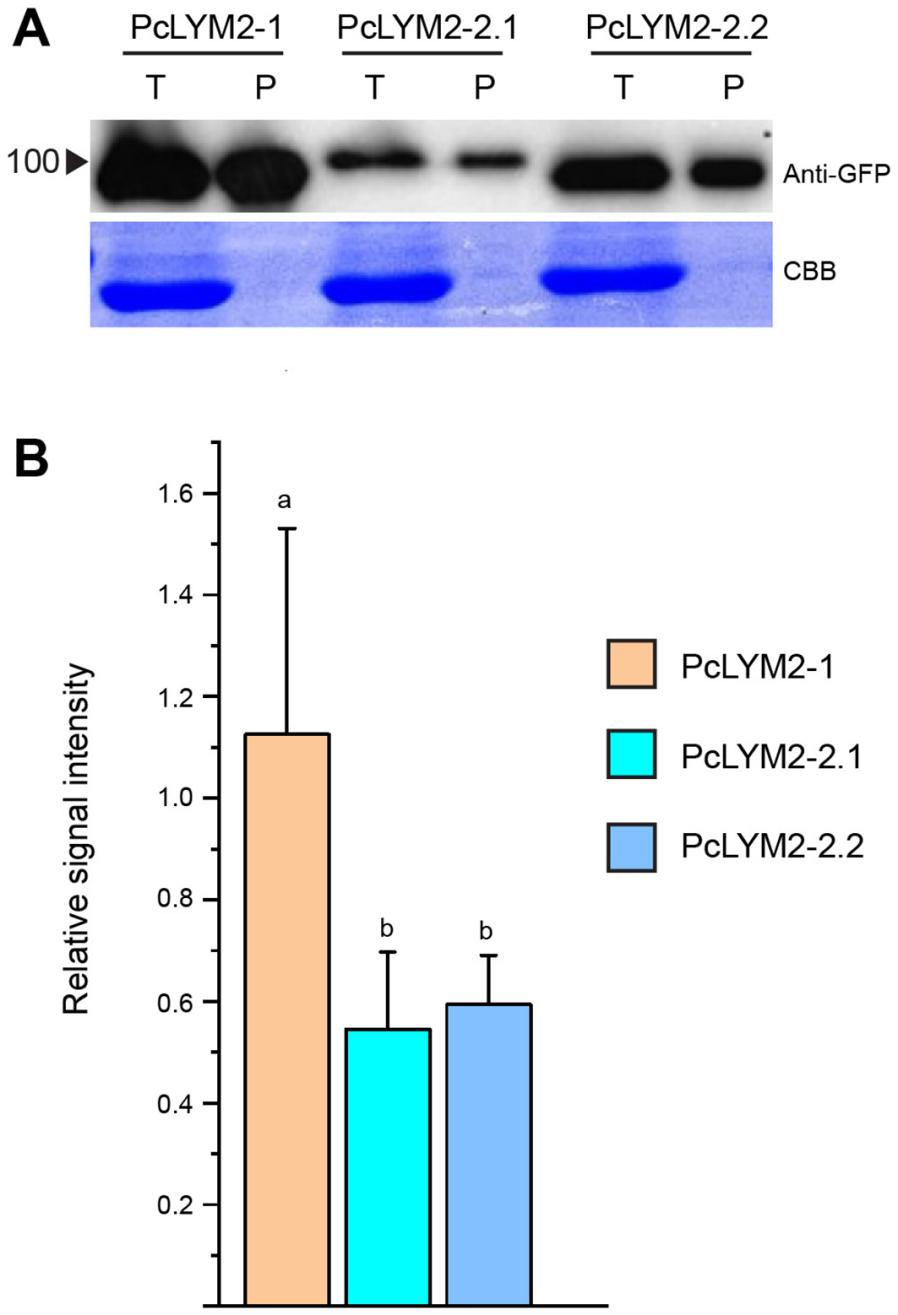
Poplar PcLYM2 proteins bind to chitin. *PcLYM2-1-mVenus*, *PcLYM2-2.1-mVenus* and *PcLYM2-2.2–mVenus* were transiently expressed in *N. benthamiana* leaves. **(A)** Western blot analysis shows the comparison of total protein extracts (T) and the chitin-binding fraction purified by chitin pull-down (P) of the LYM2 homolog PcLYM2-1, as well as the PcLYM2–2 splicing variants PcLYM2-2.1 and PcLYM2-2.2. An anti GFP antibody was used to detect the mVenus-tagged proteins. Coomassie brilliant blue staining (CBB) was used to control equal protein loading. **(B)** The signals from chitin binding proteins were normalized to the signals from total protein extracts. Statistical analysis was performed by one-way ANOVA followed by Tukey’s posthoc test (*P* ≤ 0.05). Data are means ± SD (n=4 technical repeats). The experiment was repeated twice with similar results.

Arabidopsis AtLYM2 binds chitin (Fliegmann et al., 2011; Shinya et al., 2012) and is involved in chitin-induced PD closure (Faulkner et al., 2013; Cheval et al., 2020). To characterize the proposed role of poplar LYM2 homologs in chitin perception, chitin-binding assays were performed by incubating total protein extracts from *N. benthamiana* leaves transiently expressing *P_35S_:SP-mVenus-PcLYM2-1*, *P_35S_:SP-mVenus-PcLYM2-2.1* or *P_35S_:SP-mVenus-PcLYM2-2.2* with chitin magnetic beads, allowing pull down of the bound proteins and subsequent detection and quantification using an antibody against the fluorescent marker. The amount of mVenus-PcLYM2- protein was determined via western blotting using an GFP antibody. Western blot signals of PcLYM2 in chitin magnetic bead pull-downs were normalized to signals from total protein extracts. The results indicate that all analysed PcLYM2 proteins can bind to chitin (**Figures 7A, B**).

### 3.4 PcLYM2 proteins are not involved in chitin-induced ROS burst and MAPK activation but mediate chitin-triggered PD closure

**Figure 8:**
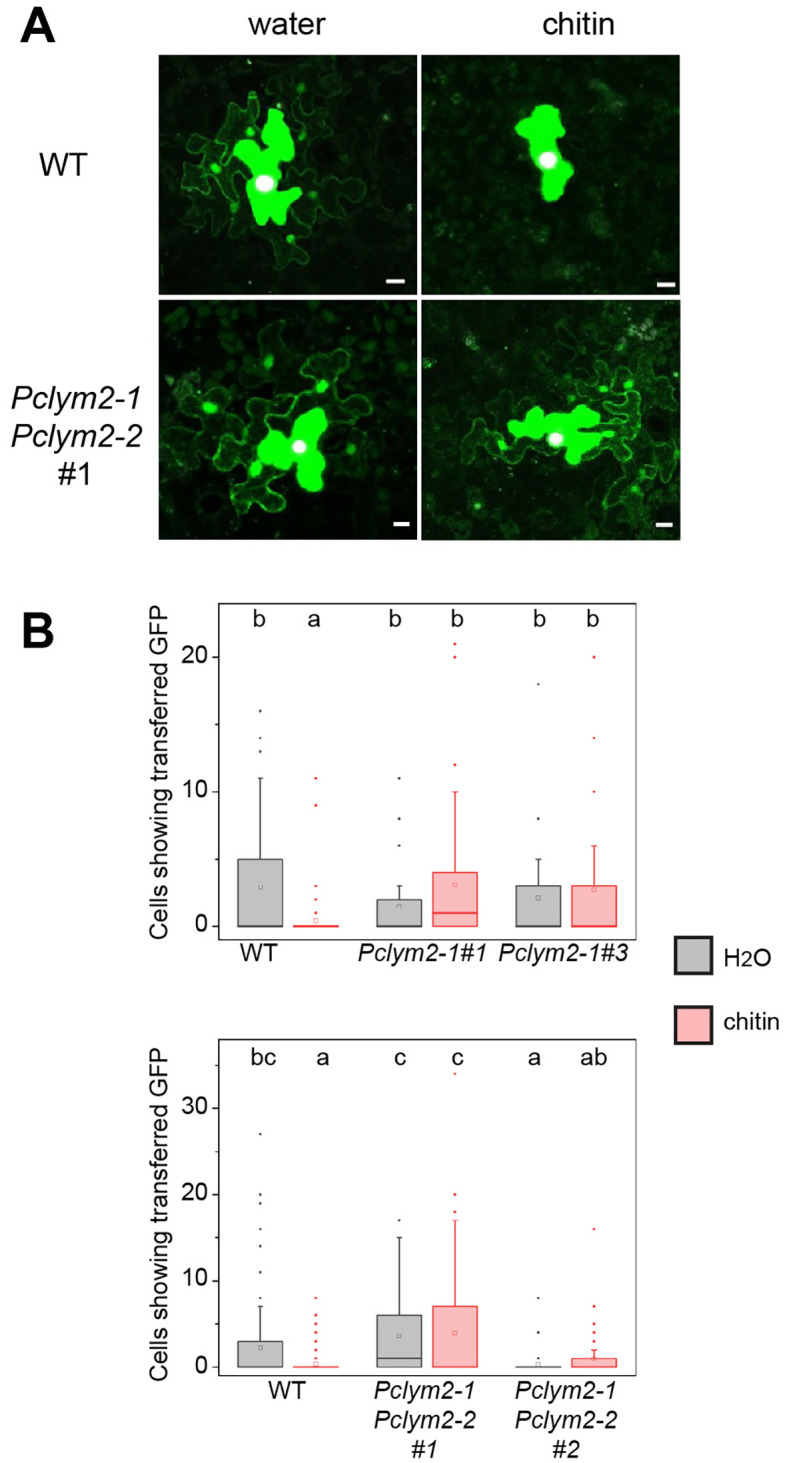
Microprojectile bombardment assays show that PcLYM2 mediates chitin-triggered PD closure in poplar. **(A)** Chitin treatment triggers PcLYM2-mediated PD closure, resulting in a reduced number of cells showing the transferred mobile eGFP marker adjacent to the bombarded cell in WT *P.* x *canescens*, but not in *Pclym2–1 Pclym2–2* double knock-out mutants. Representative results are shown. Microprojectile bombardment was done using a plasmid for expression of a mobile cytosolic *eGFP* and an immobile nuclear *mKate* marker. Images are overlays of the mKate and eGFP channel, respectively. The displayed images are a maximum projection of 10 Z-stack series taken 1 µm apart. Scale bar = 10 µm **(B)** Two *Pclym2–1* single knock-out lines (upper graph) and two *Pclym2–1 Pclym2–2* double knock-out lines (lower graph) were characterized. WT *P.* x *canescens* was included as a control. The number of adjacent cells showing transferred eGFP from the bombarded cell was counted. Counting was done three times by different persons in a blind study. Statistical analyses of counts was conducted with a generalized linear mixed-effects model using a negative binomial distribution. An Analysis of Deviance (ANODE) was applied to the model to test for significant interaction effects. The ANODE results reveal a significant interaction between the treatment and the lines (p = 3.707×10^-07^ for single knockouts, 6.643×10–^09^ for double knockouts, **Supplementary Table 1**). The Tukey’s test shows that the chitin treatment has a significant effect in wildtype plants, but not in the *PcLYM2* knockout lines.

To analyse the role of PcLYM2 proteins in poplar, knock-out lines were generated through CRISPR/Cas9 gene editing. Four sgRNAs (T1-T4) were designed to simultaneously edit both alleles of *PcLYM2–1* and *PcLYM2–2* including the splicing variants (**Supplementary Figure 2A**). Two double knock-out mutants of *PcLYM2–1* and *-2* and four single knock-out lines of *PcLYM2–1* were obtained. Editing at T1 and T2 introduced a premature stop codon or a frame shift in the coding sequence obtained (**Supplementary Figure 2A**). ROS burst and MAPK assays were performed with the knock-out lines, showing that loss of LYM2 function in poplar does not abolish these plant responses to chitin treatment (**Supplementary Figures 3A–C**). These results indicate that unlike rice CEBiP, poplar LYM2 proteins are not involved in canonical chitin-induced defense signaling. To examine whether *PcLYM2* proteins have a similar function to *AtLYM2* in mediating chitin-induced PD closure, microprojectile bombardment using a plasmid for expression of a mobile cytosolic eGFP and an immobile nuclear mKate marker was carried out with leaf discs of *PcLYM2* knock-out mutants and wildtype plants as a control. The cytosolic eGFP can diffuse through plasmodesmata to neighbouring cells, while the nuclear mKate marker stays in the nucleus and flags the bombarded cell expressing the marker genes. The numbers of adjacent cells showing eGFP signals are used as a parameter for plasmodesmal flux (Faulkner et al., 2013). A reduced number of neighboring cells showing the mobile eGFP marker was observed in the WT *P. x canescens* after chitin treatment, but not in the *PcLYM2* double knock-out mutants (**Figures 8A, B**, **Supplementary Table 1**), indicating that *PcLYM2* mediates chitin-induced PD closure similar to *AtLYM2*. Moreover, the single knock-out lines of *PcLYM2-1* also showed the same response as the double knock-out lines following chitin treatment (**Figure 8B**, **Supplementary Table 1**), indicating that PcLYM2-1 is an essential LysM-RLP in poplar leaves involved in this process.

## 4 Discussion

### 4.1 PcLYM2 proteins are GPI-anchored proteins with unique splicing variants

Two genes, *PcLYM2–1* and *PcLYM2–2* were identified as *LYM2* orthologs in *P.* x *canescens*. The presence of two gene copies is due to a whole genome-duplication in poplar (Tuskan et al., 2006; Vanneste et al., 2014). As a result of a duplication event, a genome contains paralogs of a gene. Over time, mutations may accumulate in one of the paralogs, which can result in either gene deletion/inactivation or functional modification (Mach, 2014; Vanneste et al., 2014). Both *PcLYM2* paralogs persist in the poplar genome (**Figure 1**) and our data indicate that functionality of these paralogs has been conserved (**Figures 6**, **7**). In *P.* x *canescens*, a hybrid poplar used in this study, not only two paralogs exist, but each gene has two alleles representing the parental lines *P. tremula* and *P. alba*.

Genomic, transcriptomic and mass spectrometry analyses revealed that one of the two *PcLYM2* paralogs, *PcLYM2-2*, is subject to alternative splicing which generates the protein variants PcLYM2-2.1 and PcLYM2-2.2 (**Figure 2**, **3**). Alternative splicing is a mechanism of gene regulation that may increase protein diversity or play a role in regulation of protein activity (Chaudhary et al., 2019). As a mode of alternative splicing, exon skipping, intron-retention, mutually exclusive exons, alternative donor site, alternative acceptor site and alternative polyadenylation can occur (Zhang et al., 2015; Chaudhary et al., 2019). Comparison of gene structure and cDNA sequences of *PcLYM2-2* splicing variants shows that the splicing in *PcLYM2–2* occurs through mutually exclusive splicing of exon 1 (**Figure 2A**). This alternative splicing mode is not common in plants compared to intron retention and exon skipping (Zhang et al., 2015). Mutually exclusive splicing may result in proteins with identical domain composition, but differing amino acid sequences in the domains (Chen et al., 2007). The *PcLYM2–2* alternative transcripts are translated into two full-size splicing variants that have a similar protein domain organization, but different LysM-domains at the N-terminus (**Figure 2B**). The fact that the LysM domains of PcLYM2–1 and PcLYM2-2.2 are very similar, while the LysM domain of PcLYM2-2.1 is clearly distinct, indicates that PcLYM2–1 and PcLYM2*-*2.2 perform redundant functions and that a specific function is conceivable for PcLYM2-2.1.

### 4.2 PcLYM2 proteins are chitin-binding PD-PM-localized LysM-RLPs

Subcellular localization studies of the two poplar *LYM2* paralogs including the *PcLYM2–2* splicing variants revealed that despite different amino acid compositions of the LysM domains, all identified PcLYM2 proteins reside at the same location, at both PM and PD-PM (**Figure 6**). Hence, the different amino acid compositions of the LysM domains do not affect the subcellular localization. These localization studies were performed with a heterologous expression system and results may not match the subcellular localization in poplar exactly. However, the fact that poplar LYM2 regulates chitin-induced PD closure also suggests presence of PcLYM2 in PD.

LYM2 in Arabidopsis (AtLYM2) and in rice (OsCEBiP) were shown to be involved in chitin perception but mediate different chitin-induced pathways in the two species (Kaku et al., 2006; Faulkner et al., 2013; Cheval et al., 2020). As a first approach to analyze the involvement of poplar LYM2 proteins in chitin signaling, we tested their chitin binding capacity in chitin-binding assays. In mass spectrometry analyses of chitin binding proteins from *P.* x *canescens* leaves, all characterized PcLYM2 proteins were found (**Figure 4**). Similarly, the GFP-tagged versions of all PcLYM2 proteins are capable of binding chitin (**Figure 7A**). These data indicate that also poplar LYM2 proteins play a role in chitin perception.

### 4.3 PD-localized PcLYM2 proteins mediate chitin-triggered PD closure

To analyse whether chitin perception by PcLYM2 proteins mediates primary chitin-triggered defense signaling involving ROS burst and MAPK activation or controls plasmodesmal flux, functional characterization of these two pathways was performed. Knocking out *PcLYM2* genes does not abolish chitin triggered ROS burst and MAPK activation in poplar (**Supplementary Figures 3A, B**). This suggests that PcLYM2 proteins are not crucial components of the canonical poplar chitin receptor complex as shown for OsCEBiP in rice (Kaku et al., 2006; Miya et al., 2007; Shimizu et al., 2010; Hayafune et al., 2014; Kouzai et al., 2014), but might be responsible for PD flux regulation comparable to Arabidopsis LYM2. PD flux analysis on leaves of *PcLYM2* double and single knock-out lines revealed that PcLYM2 proteins are indeed indispensable for chitin-triggered PD closure. Knockout of only *PcLYM2–1* is sufficient to abolish chitin mediated plasmodesmal flux regulation. Thus, PcLYM2–1 is likely a component of a chitin receptor complex that plays a crucial role in mediating chitin-triggered PD closure in poplar leaves. The PcLYM2-2 paralog may have specific functions in other tissues or under certain developmental or stress conditions.

The molecular binding mechanism of LysM-RLP to chitin was described in rice CEBiP, showing that OsCEBiP homodimerizes in a sandwich-type dimerization upon binding to chitin (Hayafune et al., 2014; Liu et al., 2016). Hence, dimerization between poplar LYM2 proteins may also be necessary for chitin perception, which might occur in a homo- or heterodimerization mode that includes interaction between the same or different PcLYM2 paralogs or splicing variants. The splicing variants of PcLYM2–2 show tissue-specific transcript abundances (**Figure 5**) and, as a consequence, putative dimerization of PcLYM2-2 variants with PcLYM2-1 may occur in a tissue-specific manner. In wood, PcLYM2-2.1 could be the predominant interaction partner of PcLYM2-1, while in bark and leaves the splicing variant PcLYM2-2.2 might be the main heterodimerization component. The two possible PcLYM2-1/PcLYM2–2 heterodimers may have a different chitin affinity, might mediate different downstream responses or even bind ligands other than chitin. For the canonical chitin receptor complex, consisting of CERK1, LYK4 and LYK5, it has been shown that changes in receptor composition affect signaling specificity. A receptor consisting of CERK1 and the co-receptor LYK5 is not competent to trigger sufficient ROS production, while a CERK1-LYK4 complex efficiently mediates chitin induced induction of RBOHD activity (Mittendorf et al., 2024). In addition, also homo- or heterodimerization of the two splicing variants of PcLYM2-2 is possible, for example in tissues where PcLYM2-1 is less abundant.

Apart from a potential role in allowing signalling variety via heterodimerization, PcLYM2 variants may provide protection against pathogen effectors that target plasmodesmata (Germain et al., 2018; Rahman et al., 2021) to ensure that chitin induced plasmodesmal closure stays functional after pathogen attack. In this scenario, duplication of the potential *PcLYM2* effector target gene generates a decoy with a role in the immune response, but with no function in pathogen induced plasmodesmal closure (van der Hoorn and Kamoun, 2008).

For initiating chitin-induced downstream signaling of PD closure, PcLYM2 proteins that do not have an intracellular signalling domain require association with a LysM-RLK as proposed for AtLYM2-mediated chitin-triggered PD closure in Arabidopsis. Cheval et al. (2020) propose AtLYK4 and AtLYK5 as partners of AtLYM2. Likewise, the formation of a receptor complex with PcLYM2 for chitin-induced PD closure in poplar remains to be characterized. The poplar genome encodes two *LYK4* and two *LYK5* paralogs (Cope et al., 2023), which are potential partners for PD-related chitin signaling of poplar LYM2. Defining the LYM2 receptor complex is a key question of future research on LYM2 proteins in dicots.

## Data Availability

The datasets presented in this study can be found in online repositories. The names of the repository/repositories and accession number(s) can be found below: www.ebi.ac.uk/pride/, PXD058986.
