# Supplementary material for: LYM2 mediates chitin-induced plasmodesmal flux reduction in Populus x canescens
Source: Front Plant Sci. 2026 Jul 13;17:1879338. doi: 10.3389/fpls.2026.1879338 (PMC13402948; doi:10.3389/fpls.2026.1879338)
Supplement: Supplementary file 1 [file Table1.docx]

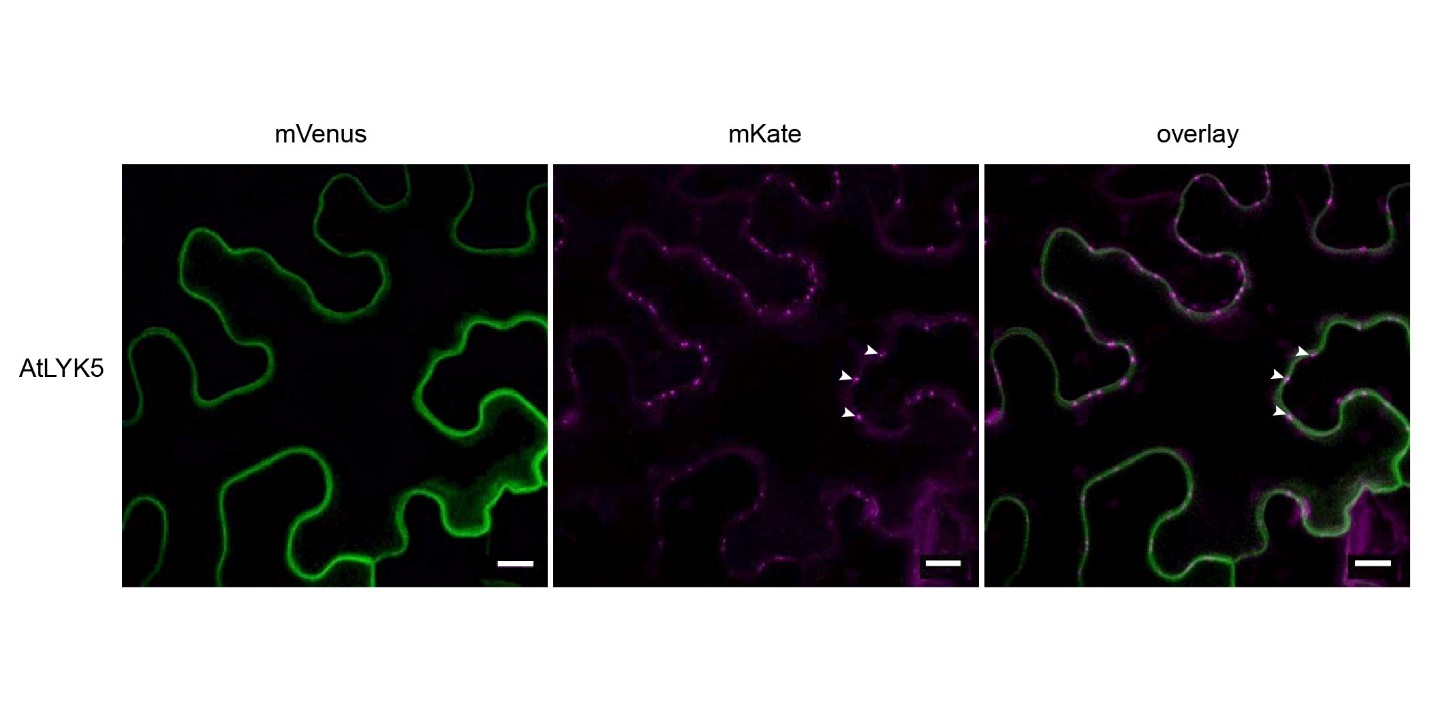


**SUPPLEMENTARY FIGURE S1**

AtLYK5 as a negative control for PD-PM localization shows even localization in the PM. Constructs expressing *AtLYK5-mVenus* and *PDLP5-mKate* were co-infiltrated into the abaxial side of *N. benthamiana* leaves. The left panel shows AtLYK5-mVenus localization in the PM. The central panel are images of PDLP5-mKate as a marker for PD-localization visible as magenta dots. In the right panel, overlay images of mVenus and mKate channels confirm the PD-PM localization of only PDLP5-mKate (indicated by arrow heads).

Scale bar = 10 µm.


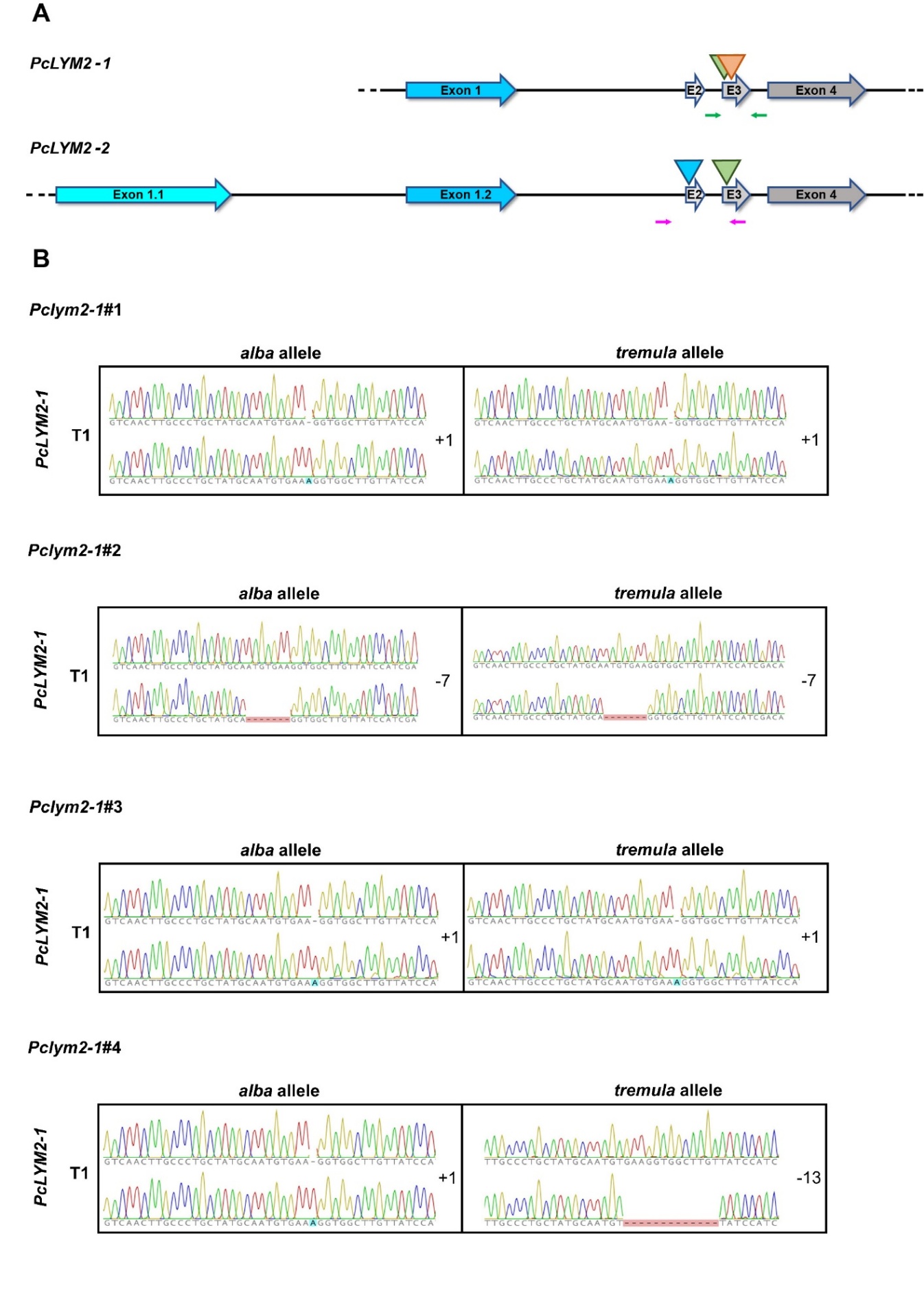


**
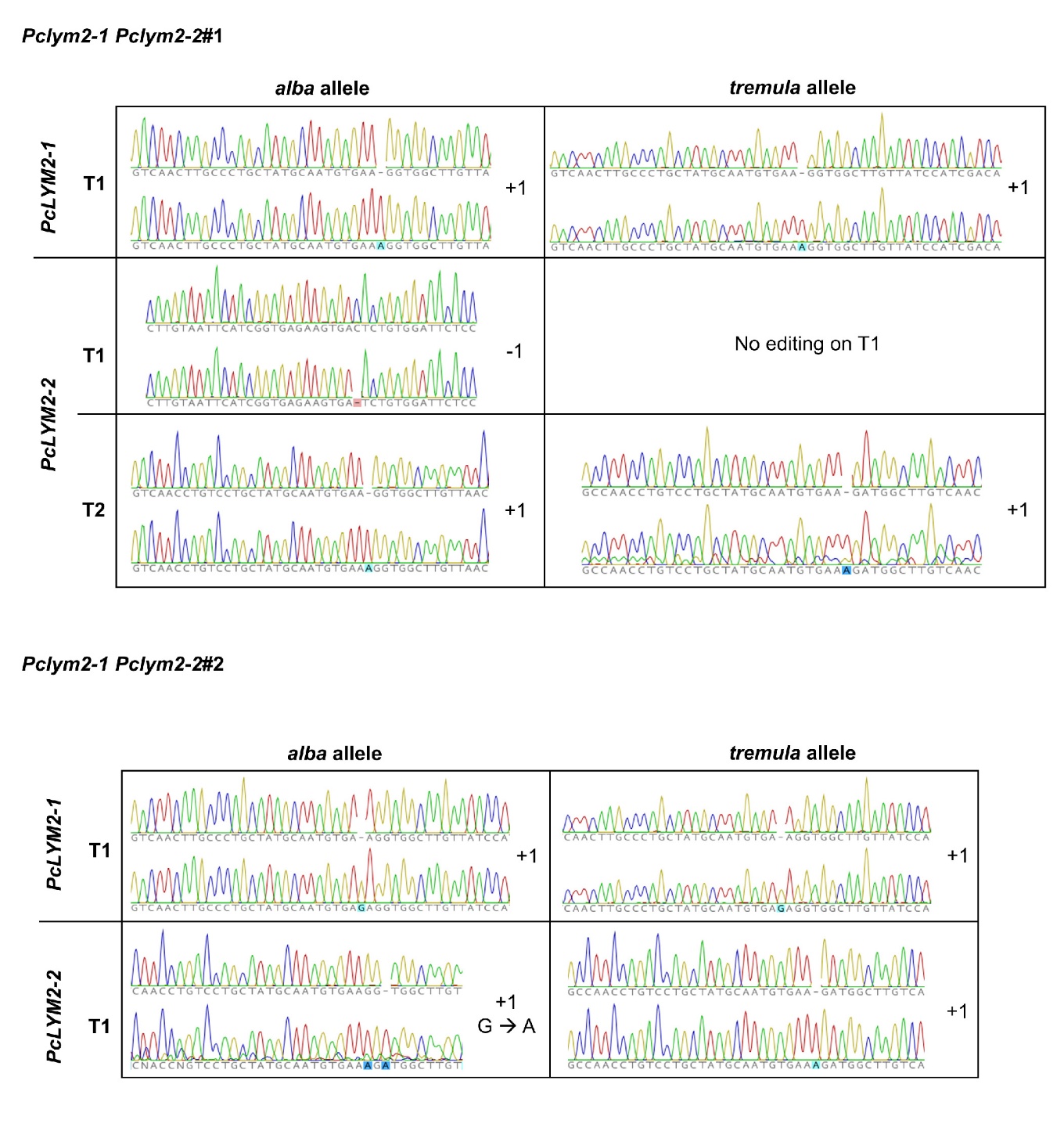
SUPPLEMENTARY FIGURE S2**

Sequence analyses of edited sites in *PcLYM2* CRISPR/Cas9 lines. **(A)** Knock-out lines of *PcLYM2* were obtained by designing sgRNAs targeting all alleles of *PcLYM2*. Targeting sites are indicated by triangles; green: T1 of *PcLYM2-1* and T2 of *PcLYM2-2* splicing variants, ochre: T2 of *PcLYM2-1*, blue: T1 of *PcLYM2-2.* Allele specific primers were used to amplify and sequence the target sites of *PcMYM2-1* (primers indicated in magenta) and *PcLYM2-2* (primers indicated in green). All editing events disrupted the function of the omega site important for GPI-anchoring. **(B)** Sequences are displayed using Chromas (Technelysium Pty Ltd, Brisbane, Australia) software. The upper sequence in each panel shows the chromatogram of wildtype *P.*x *canescens* and the lower displays the editing events in the CRISPR/Cas9 lines. Insertions, deletions and nucleotide exchanges are indicated.


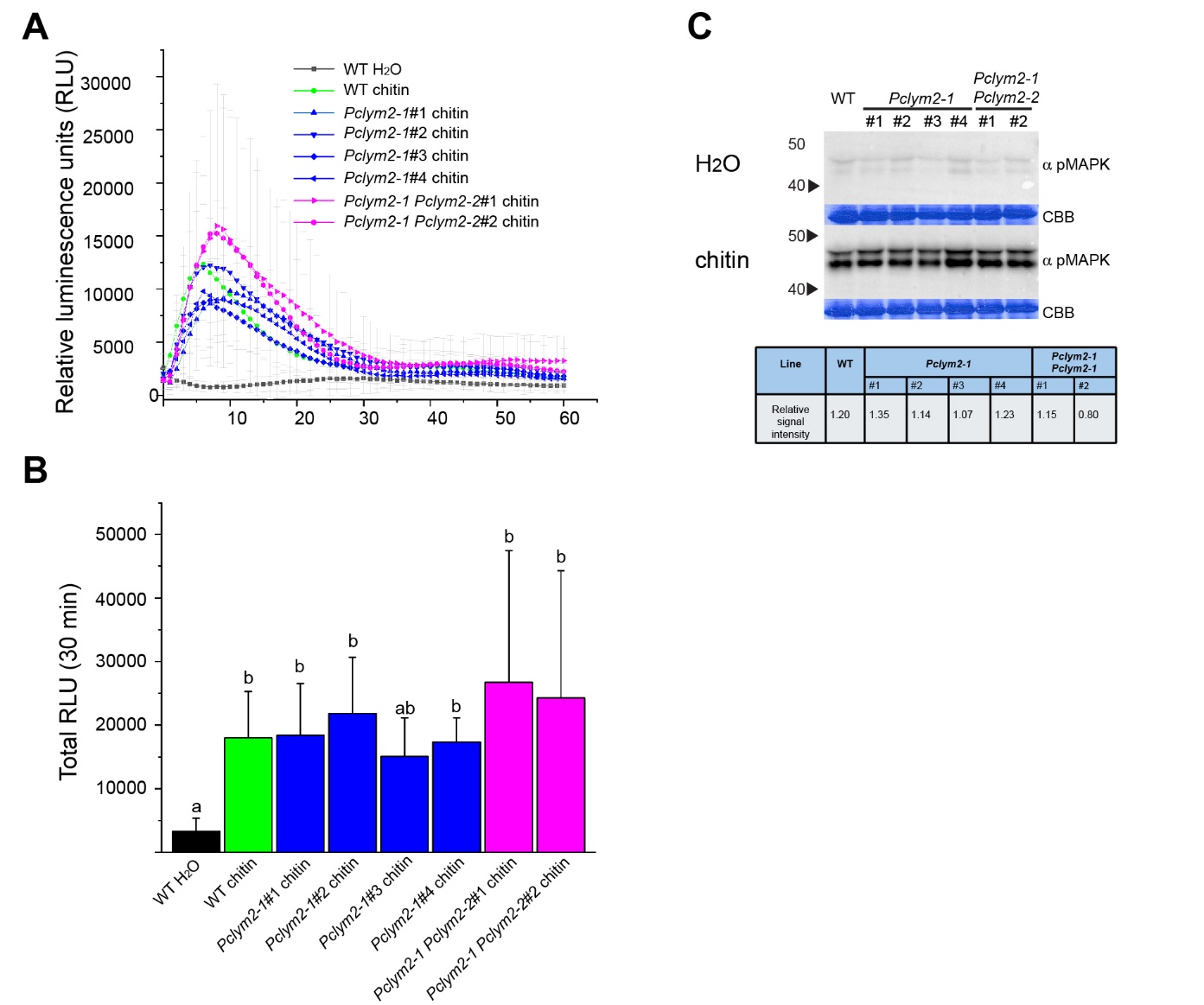


**SUPPLEMENTARY FIGURE S3**

Knock-out of *LYM2* in *P.* x *canescens* does not impair chitin induced ROS burst and MAPK activation. **(A)** For ROS burst assays leaf discs were treated with 100 µg/ml chitin. Relative luminescence units (RLU) are shown. Data are means ± SD (n = 8 biological replicates. (**B**) To quantify ROS burst data, the area under the curve (AUC) of each biological replicate was determined. For analysis the lag and the recovery phase were removed and quantification was done for a 30 min period. Two independent *Pclym2-1 Pclym2-2* double knock-out lines (marked in magenta in panel A, B) and four independent *Pclym2‑1* single knock-out lines (marked in blue in panel A, B) were tested. Wildtype plants were used as a control (marked in green in panel A, B). **(C)** For MAPK assays, leaves were infiltrated with 10 µg/ml chitin and harvested 10 minutes post infiltration. Protein samples were extracted from a pool of three plants. Coomassie brilliant blue staining (CBB) was used to control equal protein loading. The table shows signal intensities of chitin treated samples normalized to the loading control (CBB). Each experiment was independently repeated twice showing similar results.
