# Supplementary material for: LYM2 mediates chitin-induced plasmodesmal flux reduction in Populus x canescens
Source: Front Plant Sci. 2026 Jul 13;17:1879338. doi: 10.3389/fpls.2026.1879338 (PMC13402948; doi:10.3389/fpls.2026.1879338)
Supplement: Supplementary file 2 [file Table2.docx]

**SUPPLEMENTARY TABLE S1**

PcLYM2 mediates chitin-triggered PD closure in poplar*.*To analyse the data displayed in Figure 8 B we conducted statistical analyses of counts with a generalized linear mixed-effects model using a negative binomial distribution. An Analysis of Deviance (ANODE) was applied to the model to test for significant interaction effects.

*PcLYM2* single knockouts

Analysis of Deviance Table (Type II tests)

Response: counts

LR Chisq Df (Pr>Chisq)

line 10.591 2 0.005013 **

treatment 14.613 1 0.000132 ***

line:treatment 29.616 2 3.707e-07 ***

Significance codes codes: 0 ‘***’ 0.001 ‘**’ 0.01 ‘*’

*PcLYM2* double knockouts

Analysis of Deviance Table (Type II tests)

Response: counts

LR Chisq Df (Pr>Chisq)

line 55.113 2 1.077e-12 ***

treatment 7.352 1 0.006697 **

line:treatment 37.660 2 6.643e-09 ***

Significance codes codes: 0 ‘***’ 0.001 ‘**’ 0.01 ‘*’
