# Supplementary material for: LYM2 mediates chitin-induced plasmodesmal flux reduction in Populus x canescens
Source: Front Plant Sci. 2026 Jul 13;17:1879338. doi: 10.3389/fpls.2026.1879338 (PMC13402948; doi:10.3389/fpls.2026.1879338)
Supplement: Supplementary file 3 [file Table3.docx]

**SUPPLEMENTARY TABLE S2:** Oligonucleotides

| Primer Name | Function | Sequence (5‘ to 3‘) |
| --- | --- | --- |
| F-183500 | Forward primer for amplification of *PcLYM2-1* from leaf-derived cDNA. | GCAAACGACCCAGGAATC |
| R-183500 | Reverse primer for amplification of *PcLYM2-1* from leaf-derived cDNA. | GCCCCAACATCAAGAAACAG |
| F1-143300 | Forward primer for amplification of *PcLYM2-2.1* from leaf-derived cDNA. | GGAAAGCCGACATCTCTCCG |
| R-143300 | Reverse primer for amplification of *PcLYM2-2.1* from leaf-derived cDNA (EcoRI restriction site in red). | GACGAATTCCACCCAGAGAGAAGCACC |
| F_PtremLYM2-2.1 | Forward primer for amplification of *PcLYM2-2.1* *P. tremula* allele from leaf-derived cDNA. | CAAAGCCCAACTTTTCCCAG |
| PtreLYM2-2_KO_R | Reverse primer for amplification of *PcLYM2-2.1* *P. tremula* allele from leaf-derived cDNA. | GAGAAGCACCGTTTCTTACTAG |
| F-143300.2 | Forward primer for amplification of *PcLYM2-2.2* from leaf-derived cDNA. | GTAATAACACAACAGCCAACC |
| R-143300 | Reverse primer for amplification of *PcLYM2-2.2* from leaf-derived cDNA (EcoRI restriction site in red). | GACGAATTCCACCCAGAGAGAAGCACC |
| F_PtremLYM2-2.2 | Forward primer for amplification of *PcLYM2-2.2* *P. tremula* allele from leaf-derived cDNA. | GAATTCCCCAGTTTATCCC |
| R_PtrexaLYM2-2.2 | Reverse primer for amplification of *PcLYM2-2.2* *P. tremula* and *P. alba* alleles from leaf-derived cDNA. | GATTTCTCATATGACCCAATATC |
| F_PcLYM2-2_Exon1CDS | Forward primer for amplification of exon 1 of *PcLYM2-2.1* and *PcLYM2-2.2* from gDNA (to analyze the exon-intron structure of the *PcLYM2* gene on gDNA level) | ATGGGGTTTGCCATAATTCTAATG |

| R_PcLYM2-2.1_Full | Reverse primer for amplification of *PcLYM2-2.1* from gDNA (to analyze the exon-intron structure of the *PcLYM2* gene on gDNA level) | CCAATCCTTGAAGCGAAATTGC |
| --- | --- | --- |
| M13_F | Forward primer for sequencing of the gDNA-isolated *PcLYM2-2* on the PCR 2.1 TA cloning vector (to analyze the exon-intron structure of the *PcLYM2* gene on gDNA level) | CGTTGTAAAACGACGGCCAGT |
| M13_R | Reverse primer for sequencing of the gDNA-isolated *PcLYM2-2* on the PCR 2.1 TA cloning vector (to analyze the exon-intron structure of the *PcLYM2* gene on gDNA level) | CAGGAAACAGCTATGACCATG |
| F-143300.1 | Internal forward primer (designed on the exon1) for sequencing of gDNA-isolated *PcLYM2-2.1* (to analyze the exon-intron structure of the *PcLYM2* gene on gDNA level) | GTGGAAGAAGGGAGCACCG |
| F_PcLYM2-2.1_Intron | Internal forward primer (designed on the intron) for sequencing of gDNA-isolated *PcLYM2-2.1* (to analyze the exon-intron structure of the *PcLYM2* gene on gDNA level) | GCTCTAAAGCTTACGAAGATTTAAGAG |
| R_PcLYM2-2.1_Intron | Internal reverse primer (designed on the intron) for sequencing of gDNA-isolated *PcLYM2-2.1* (to analyze the exon-intron structure of the *PcLYM2* gene on gDNA level) | GCACATTGCCTAGCACTATC |
| Potri.004G183500.1_qPCR_F | Forward primer for qPCR of *PcLYM2-1* | CTCCCAAGGAATCACACCATAGC |
| Potri.004G183500.1_qPCR_R | Reverse primer for qPCR of *PcLYM2-1* | GATCTTGAGTGGAGCGTGGAG |
| Potri.009G143300.1_qPCR_F | Forward primer for qPCR of *PcLYM2‑2.1* | CAAGCACGACACACCTCCAC |
| Potri.009G143300.1_qPCR_R | Reverse primer for qPCR of *PcLYM2‑2.1* | GCTGGGCTACTGATATGGTGG |
| Potri.009G143300.2_qPCR_F | Forward primer for qPCR of *PcLYM2‑2.2* | GAAATCACACCAGAGCCAAAATGG |
| Potri.009G143300.2_qPCR_R | Reverse primer for qPCR of *PcLYM2-2.2* | GGATGAAGATTTTGTGGGCAGTG |

| Primers for amplification of gRNA and promoter fragments to generate *PcLYM2* knock-out lines | | |
| --- | --- | --- |
| gRNA_LYM2-2_T1_F | Forward primer for amplification of gRNA fragment, carrying the protospacer as 5' overhang for gRNA targeting *PcLYM2-2* (target 1/ T1: *PcLYM2-2* including the two alleles of two variants, *PcLYM2-2.1* and *PcLYM2-2.2*). | TCGGTGAGAAGTGACTCTGGTTTTAGAGCTAGAAATAGCAAGTTAAA |
| U3d_LYM2-2_T1_R | Reverse primer for amplification of U3d promoter, carrying 5' overhang of the reverse complement of the protospacer for gRNA targeting *PcLYM2-2* (T1 of *PcYLM2-2*). | CAGAGTCACTTCTCACCGATGACCAATGGTGCTTTGTAG |
| gRNA_LYM2_T1/T2_F | Forward primer for amplification of gRNA fragment, carrying protospacer as 5' overhang for gRNA targeting two *LYM2* orthologs (target 1/ T1 for *PcLYM2-1,* but assigned as T2 for *PcLYM2-2*, including two variants and both alleles). | CCTGCTATGCAATGTGAAGGGTTTTAGAGCTAGAAATAGCAAGTTAAA |
| U3b_LYM2_T1/T2_R | Reverse primer for amplification of U3b promoter, carrying the reverse complement of the protospacer as 5' overhang for gRNA targeting two *LYM2* orthologs (T1 of *PcLYM2-1*, and assigned as T2 of *PcLYM2-2*). | CCTTCACATTGCATAGCAGGTGACCAATGTTGCTCCCTC |
| gRNA_LYM2-1_T2_F | Forward primer for amplification of gRNA fragment, carrying protospacer as 5' overhang for gRNA targeting *PcLYM2-1* (target 2/ T2: *PcLYM2‑1* both alleles). | TCTTCACAACTCTCGCCACAGTTTTAGAGCTAGAAATAGCAAGTTAAA |

| U6-1_LYM2-1_T2_R | | | | | Reverse primer for amplification of U6-1 promoter, carrying 5' overhang of the reverse complement of the protospacer for gRNA targeting both alleles of *PcLYM2-1* (T2 of *PcLYM2-1*). | TGTGGCGAGAGTTGTGAAGACAATCACTACTTCGTCTCTAACC | | | |
| --- | --- | --- | --- | --- | --- | --- | --- | --- | --- |
| gRNA_LYM2-1_T3_F | | | | | Forward primer for amplification of gRNA fragment, carrying protospacer as 5' overhang for gRNA targeting the 2nd allele of *PcLYM2-1* (target 3/ T3: *PcLYM2-1* allele 1) | CCTGCAATGCAATGTGAAGGGTTTTAGAGCTAGAAATAGCAAGTTAAA | | | |
| U6-29_LYM2-1_T3_R | | | | | Reverse primer for amplification of U6-1 promoter, carrying 5' overhang of the reverse complement of the protospacer for gRNA targeting allele 1 of *PcLYM2-1* (T3). | CCTTCACATTGCATTGCAGGCAATCTCTTAGTCGACTCTACCA | | | |
| Primer used to assemble sgRNA fragment into the pYLCRISPR/Cas9P_35S_-N vector | | | | | | | | | |
| Pgs_GA3 | | | | | Reverse primer for the second gRNA expression cassette, used in 2^nd^ PCR (overlap extension) as outer primer. Introduces Gibson assembly site 2 for fusion with the third gRNA cassette. Design by Ma et al., 2015. | CCACGCATACGATTTAGGTGACACTATAGCGCATCCACTCCAAGCTCTTG | | | |
| U_GA3 | | | | | Forward primer for the third gRNA expression cassette, used in 2^nd^ PCR (overlap extension) as outer primer. Introduces Gibson assembly site 2 for fusion with the second gRNA cassette. Design by Ma et al., 2015. | CGCTATAGTGTCACCTAAATCGTATGCGTGGTGGAATCGGCAGCAAAGG | | | |
| Pgs_GA4 | | | | | Reverse primer for the third gRNA expression cassette, used in 2^nd^ PCR (overlap extension) as outer primer. Introduces Gibson assembly site 2 for fusion with fourth gRNA cassette. Design by Ma et al., 2015. | GTCGCTAGTTATTGCTCAGCGGCCAAGCTCATCCACTCCAAGCTCTTG | | | |
| U_GA4 | | | | | Forward primer for the fourth gRNA expression cassette, used in 2^nd^ PCR (overlap extension) as outer primer. Introduces Gibson assembly site 2 for fusion with the third gRNA cassette. Design by Ma et al., 2015. | GAGCTTGGCCGCTGAGCAATAACTAGCGACTGGAATCGGCAGCAAAGG | | | |
| Pgs_GG3 | | | | Reverse primer for the second gRNA expression cassette, used in 2^nd^ PCR (overlap extension) as outer primer. Introduces BsaI site (lowercase)-cutting non palindromic sequence and adapter sequence for cloning site 2 for ligation with the second cassette for Golden gate cloning. Design by Ma et al., 2015. | | AGCGTGggtctcGtcttcacTCCATCCACTCCAAGCTC | | | |
| Pps_GG3 | | | | Forward primer for the third gRNA expression cassette, used in 2^nd^ PCR (overlap extension) as outer primer. Introduces BsaI site (lowercase)-cutting non palindromic sequence and adapter sequence for cloning site 2 for ligation with second cassette for Golden gate cloning. Design by Ma et al., 2015. | | TTCAGAggtctcTaagacttTGGAATCGGCAGCAAAGG | | | |
| Pgs_GG4 | | | Reverse primer for the third gRNA expression cassette, used in 2^nd^ PCR (overlap extension) as outer primer. Introduces BsaI site (lowercase)-cutting non palindromic sequence and adapter sequence for cloning site 2 for ligation with the fourth cassette for Golden gate cloning. Design by Ma et al., 2015. | | | | AGCGTGggtctcGagtccttTCCATCCACTCCAAGCTC | | |
| Pps_GG4 | | | Forward primer for the fourth gRNA expression cassette, used in 2^nd^ PCR (overlap extension) as outer primer. Introduces BsaI site (lowercase)-cutting non palindromic sequence and adapter sequence for cloning site 2 for ligation with third cassette for Golden gate cloning. Design by Ma et al., 2015. | | | | TTCAGAggtctcTgactacaTGGAATCGGCAGCAAAGG | | |
| U-F | | | Forward primer for U3/U6 promoters. Design by Ma et al., 2015. Used in the first round of overlapping PCR. Reverse primer U#T# carries specific gRNA sequence used as complementary overhang. | | | | CTCCGTTTTACCTGTGGAATCG | | |
| gR-R | Reverse primer for gRNA. Design by Ma et al., 2015. Used in the first round of overlapping PCR. Forward primer  gR-T# carries specific gRNA sequence used as complementary overhang. | | | | | | | | CGGAGGAAAATTCCATCCAC |
| Pps-GGL | Forward primer for the first gRNA expression cassette, used in 2^nd^ PCR (overlap extension) as outer primer. Introduces BsaI site (lowercase)-cutting non palindromic sequence and adapter sequence for cloning site B-L in destination vector pYLCRISPR/Cas9P35S-N. Additionally introduces SpeI site (UPPERCASE). Design by Ma et al., 2015. | | | | | | | | TTCAGAggtctcT**ctcg**ACTAGTATGGAATCGGCAGCAAAGG |
| Pgs-GG2 | Reverse primer for the first gRNA expression cassette, used in 2^nd^ PCR (overlap extension) as outer primer. Introduces BsaI site (lowercase)-cutting non palindromic sequence and adapter sequence for cloning site 2 for ligation with 2^nd^ cassette. Design by Ma et al., 2015. | | | | | | | | AGCGTGggtctcGtcagggTCCATCCACTCCAAGCTC |
| Pps-GG2 | Forward primer for the second gRNA expression cassette, used in 2^nd^ PCR (overlap extension) as outer primer. Introduces BsaI site (lowercase)-cutting non palindromic sequence and adapter sequence for cloning site 2 for ligation with 1^st^ cassette. Design by Ma et al., 2015. | | | | | | | | TTCAGAggtctcT**ctga**cacTGGAATCGGCAGCAAAGG |
| Pgs-GGR | | Reverse primer for the last gRNA expression cassette, used in 2^nd^ PCR (overlap extension) as outer primer. Introduces BsaI site (lowercase)-cutting non palindromic sequence and adapter sequence for cloning site B-R in destination vector pYLCRISPR/Cas9P35S-N. Additionally introduces MluI site (UPPERCASE). Design by Ma et al., 2015. | | | | | | AGCGTGggtctcGaccgACGCGTATCCATCCACTCCAAGCTC | |

| Primer pairs for amplification of the target sites for validation of editing | | | | |
| --- | --- | --- | --- | --- |
| F-LYM2-1 alba | Forward primer specific to amplify allele of CRISPR *LYM2-1* target site (Designed based on genomic sequence of *P. alba* allele Potri.004G183500). | | | GGGTTGAGCTACAGGCTAC |
| R_CRISPR_LYM2-1_Seq | Reverse primer to check the edited sequence of CRISPR construct specific for *LYM2-1* (both alleles). Designed on the 3rd intron. | | | CGAAGAATACTACATGCAGC |
| F-LYM2-1 tremula (2) | Forward primer specific to amplify allele of CRISPR *LYM2-1* target site (Designed based on genomic sequence of *P. tremula* allele Potri.004G183500). | | | GGGATGAGCTACAGGCTAT |
| R_CRISPR_LYM2-1_Seq | Reverse primer to check the edited sequence of CRISPR construct specific for *LYM2-1* (both alleles). Designed on the 3rd intron. | | | CGAAGAATACTACATGCAGC |
| F_CRISPR_LYM2-2_Seq | Forward primer to check the edited sequence of CRISPR construct specific for *LYM2-2* (both variants including the two alleles from each). Designed on the 1st intron. | | | GTAGCTGTCTTCAAATGTTGG |
| R_CRISPR_LYM2-2_Seq | Reverse primer to check the edited sequence of CRISPR construct specific for *LYM2-2* (both variants including the two alleles from each). Designed on the 3rd exon. | | | GGCTAGAGTTGTGAAGATCC |
| PtreLYM2-2_KO_F | | Forward primer specific to amplify allele of CRISPR *LYM2-2* target site (Designed based on genomic sequence of *P. tremula* allele Potri.009G143. | AGGCTCGTTGGCATTAAG | |
| PtreLYM2-2_KO_R | | Reverse primer specific to amplify allele of CRISPR *LYM2-2* target site (Designed based on genomic sequence of *P. tremula* allele Potri.009G14. | GAGAAGCACCGTTTCTTACTAG | |

| Primers to generate 35S:mVenus_*PcLYM2-1* genetic construct | | | | | | |  |
| --- | --- | --- | --- | --- | --- | --- | --- |
| p35S_F_PcLYM2-1 | Forward primer for amplification of promoter 35S from donor plasmid (pHG141), carrying 5' overhang of the destination vector backbone. | | | | | GAATTGGGTACCGGCGCGGGTCCCCAGATTAGCCTTTTCAATTTC |  |
| R_p35S_PcLYM2 | | Reverse primer for amplification of promoter 35S from donor plasmid (pHG141). | | GGATCCCGGACCGCGGTC | | |  |
| F_SP_PcLYM2-1 | | Forward primer for amplification of *PcLYM2-1* signal peptide, carrying 5’ overhang of the 3’ end of p35S fragment. | | GACCGCGGTCCGGGATCCATGGGTTTCCATTTCACTTCGC | | |  |
| R_SP_PcLYM2-1 | | Reverse primer for amplification of *PcLYM2-1* signal peptide. | | GGAAGAAGATCTTGAGTGGAGC | | |  |
| Venus_F_PcLYM2-1 | | | Forward primer for amplification of Venus coding sequence (CDS) from donor plasmid (pHG151), carrying 5' overhang of the 3’ end of *PcLYM2-1* signal peptide. | | GCTCCACTCAAGATCTTCTTCCATGGTGAGCAAGGGCGAG | | |
| Venus_R_PcLYM2 | | | Reverse primer for amplification of Venus fluorescent tag from donor plasmid (pHG151). | | GTACAGCTCGTCCATGCC | | |
| F_PcLYM2-1-CDS | | | Forward primer for amplification of *PcLYM2-1* CDS, carrying 5' overhang of the 3’ end of Venus fluorescent tag with linker. | | GGCATGGACGAGCTGTACAAGCACGCGGTGATGCAGACCTTCAAATGTAGCAC | | |

| R_PcLYM2-1-CDS | Reverse primer for amplification of *PcLYM2-1* CDS, carrying 5' overhang of the destination vector backbone. | GAACTAGTGGATCCCCCGGGTCAAAGAAGATACACAAGAAGAAG |
| --- | --- | --- |
| F_JE37Sulf_InversePCR | Forward primer for inverse PCR to amplify the destination vector (modified JE#37 with Sulfadiazine resistance marker). | CCCGGGGGATCCACTAGTTC |
| R_JE37Sulf_InversePCR | Reverse primer for inverse PCR to amplify the destination vector (modified JE#37 with Sulfadiazine resistance marker). | CGCGCCGGTACCCAATTC |
| Primers to generate 35S:mVenus_*PcLYM2-2.1* | | |
| p35S_F_PcLYM2-1 | Forward primer for amplification of promoter 35S, signal peptide of *PcLYM2-2.1* and mVenus in the respective order from the donor vector. | GAATTGGGTACCGGCGCGGGTCCCCAGATTAGCCTTTTCAATTTC |
| Venus_R_PcLYM2-2.1 | Reverse primer for amplification of promoter 35S, signal peptide of *PcLYM2-2.1* and mVenus in the respective order from the donor vector. | GTACAGCTCGTCCATGCC |
| PcLYM2-2.1Backbone1_F | Forward primer for amplification of *PcLYM2-2.1* CDS, carrying 5' overhang of the 3’ end of Venus fluorescent tag with linker. | GGCATGGACGAGCTGTACAAGCACGCGGTGATGCAGCAAGCATTCAAGTGC |

| G1_PcLYM2-2.1LysM3_R | Reverse primer for amplification of *PcLYM2-2.1* CDS, carrying 5' overhang of the destination vector backbone. | GAACTAGTGGATCCCCCGGGTTAAAGAAGATATACAAGAAGCAGAATC |
| --- | --- | --- |
| R_JE37Sulf_InversePCR | Forward primer for inverse PCR to amplify the destination vector (modified JE#37 with Sulfadiazine resistance marker). | CGCGCCGGTACCCAATTC |
| F_JE37Sulf_InversePCR | Reverse primer for inverse PCR to amplify the destination vector (modified JE#37 with Sulfadiazine resistance marker). | CCCGGGGGATCCACTAGTTC |
| Primers to generate 35S:mVenus_*PcLYM2-2.2* genetic construct | | |
| F_SP_PcLYM2-2.2 | Forward primer for amplification of *PcLYM2-2.2* signal peptide, carrying 5’ overhang of the 3’ end of p35S fragment. | GACCGCGGTCCGGGATCCATGGGTTTCCATTTCATTC |
| R_SP_PcLYM2-2.2 | Reverse primer for amplification of *PcLYM2-2.2* signal peptide. | GGATGAAGATTTTGTGGGC |
| F_Venus_PcLYM2-2.2 | Forward primer for amplification of Venus CDS from donor plasmid (pHG151), carrying 5' overhang of the 3’ end of *PcLYM2-2.2* signal peptide. | GCCCACAAAATCTTCATCCATGGTGAGCAAGGGCGAG |
| R_Venus_PcLYM2-2.2 | Reverse primer for amplification of Venus fluorescent tag from donor plasmid (pHG151). | GTACAGCTCGTCCATGCC |

| F_PcLYM2-2.2_CDS2 | Forward primer for amplification of *PcLYM2-2.2* CDS, carrying 5' overhang of the 3’ end of Venus fluorescent tag with linker. | | GGCATGGACGAGCTGTACAAGCACGCGGTGATGCAAACCTTCAAATGTAGCTCACC | |
| --- | --- | --- | --- | --- |
| R_PcLYM2-2.2_CDS2 | Reverse primer for amplification of *PcLYM2-2.2* CDS. | | TTAAAGAAGATATACAAGAAGCAGAATC | |
| F_T35S_PcLYM2-2.2 | | Forward primer for amplification of terminator 35S from donor plasmid (pHG141), carrying 5’ overhang of the 3’ end of PcLYM2-2.2 CDS. | | GATTCTGCTTCTTGTATATCTTCTTTAATCTAGAGTCCGCAAAAATCACC |
| R_T35S_PcLYM2-2 | | Reverse primer for amplification of terminator 35S from donor plasmid (pHG141), carrying 5' overhang of the destination vector backbone. | | CTATAGGGCGAATTGGGTACCGGTCACTGGATTTTGGTTTTAGG |
| R_JE37Sulf_InversePCR | | Forward primer for inverse PCR to amplify the destination vector with Sulfadiazine resistance marker). | | CGCGCCGGTACCCAATTC |
| F_JE37Sulf_InversePCR | | Reverse primer for inverse PCR to amplify the destination vector with Sulfadiazine resistance marker). | | CCCGGGGGATCCACTAGTTC |
